# Exploring the eukaryotic Yip and REEP/Yop superfamily of membrane-shaping adapter proteins (MSAPs): A cacophony or harmony of structure and function?

**DOI:** 10.3389/fmolb.2022.912848

**Published:** 2022-08-19

**Authors:** Timothy Angelotti

**Affiliations:** Department of Anesthesiology, Perioperative and Pain Medicine Stanford University Medical School, Stanford, CA, United States

**Keywords:** endoplasmic reticulum, golgi, hereditary spastic paraplegia, membrane-shaping adapter protein, YIPF, Yif, REEP, PRAF

## Abstract

Polytopic cargo proteins are synthesized and exported along the secretory pathway from the endoplasmic reticulum (ER), through the Golgi apparatus, with eventual insertion into the plasma membrane (PM). While searching for proteins that could enhance cell surface expression of olfactory receptors, a new family of proteins termed “receptor expression-enhancing proteins” or REEPs were identified. These membrane-shaping hairpin proteins serve as adapters, interacting with intracellular transport machinery, to regulate cargo protein trafficking. However, REEPs belong to a larger family of proteins, the Yip (Ypt-interacting protein) family, conserved in yeast and higher eukaryotes. To date, eighteen mammalian Yip family members, divided into four subfamilies (Yipf, REEP, Yif, and PRAF), have been identified. Yeast research has revealed many intriguing aspects of yeast Yip function, functions that have not completely been explored with mammalian Yip family members. This review and analysis will clarify the different Yip family nomenclature that have encumbered prior comparisons between yeast, plants, and eukaryotic family members, to provide a more complete understanding of their interacting proteins, membrane topology, organelle localization, and role as regulators of cargo trafficking and localization. In addition, the biological role of membrane shaping and sensing hairpin and amphipathic helical domains of various Yip proteins and their potential cellular functions will be described. Lastly, this review will discuss the concept of Yip proteins as members of a larger superfamily of membrane-shaping adapter proteins (MSAPs), proteins that both shape membranes via membrane-sensing and hairpin insertion, and well as act as adapters for protein-protein interactions. MSAPs are defined by their localization to specific membranes, ability to alter membrane structure, interactions with other proteins via specific domains, and specific interactions/effects on cargo proteins.

## 1 Introduction

Regulation of synthesis and export of cargo proteins with multiple transmembrane spanning domains has been studied with multiple GPCR family members and neurotransmitter transporters, revealing that ER retention/export signals, various chaperone/escort proteins, and recently, intracellular membrane-shaping adapter proteins have regulatory roles in transmembrane cargo protein trafficking (Butchbach et al., 2002; Duvernay et al., 2005; Dong et al., 2007; Ruggiero et al., 2008; Björk et al., 2013). This cellular pathway consists of multiple complex processes and mechanisms to insure proper folding, assembly, quality control, selective retention, and selective transport (Ellgaard and Helenius, 2003). Transmembrane cargo proteins are synthesized in the endoplasmic reticulum (ER), where they are folded and assembled, eventually exiting the ER after they are sorted from ER-resident proteins, to be transported through the secretory pathway to the plasma membrane (PM). They are selectively enriched into coat protein complex II (COPII) transport vesicles by the action of Sec24, based upon ER export signals, where they traverse the Golgi network for glycolytic processing and eventual transport to their membrane localizations (Pagano et al., 1999; Antonny and Schekman, 2001; Barlowe, 2003). Proper export of GPCRs and transporters is dependent upon several factors including folding rates and assembly, which may be modified by pharmacological and/or protein chaperones, as well as specific sequences within the protein that dictate ER or Golgi export or retention (Schulein et al., 1998; Hermosilla and Schülein, 2001; Petaja-Repo et al., 2002; Brothers et al., 2006; Duvernay et al., 2009). Recently, inefficient protein folding and processing has been shown to regulate PM expression of some GPCRs, with glycosylation playing a major role (Angelotti et al., 2010; Hurt et al., 2013).

While searching for proteins that could enhance heterologous cell surface expression of olfactory receptors (OR), a subset of GPCRs, a new family of six proteins termed “receptor expression-enhancing proteins” or REEPs were identified (Saito et al., 2004). Furthermore, co-expression of REEP1 led to enhanced functional surface expression for some, but not all ORs, as well as several GPCRs, including taste (TR) and α2C adrenergic receptors (α2C AR) (Behrens et al., 2006; Ilegems et al., 2010; Björk et al., 2013). These findings lead to the hypothesis that REEPs enhanced expression of a variety of poorly expressed GPCRs, possibly as chaperones or co-receptors. However, a sequence comparison revealed that REEPs are homologous to yeast (Yop1p) and barley (HVA22) proteins (Saito et al., 2004); REEPs have been alternatively named the Yip2 family (Pfeffer and Aivazian, 2004). Our understanding of REEP function is based in part on their similarity to Yop1p and HVA22, however the yeast literature has suggested a variety of unexplored intracellular functions for REEPs.

In fact, REEPs, Yop1p, and HVA22 are actually part of a much larger family of proteins, the Yip (Ypt-interacting protein) family, conserved in yeast and higher eukaryotes (Pfeffer and Aivazian, 2004). Ypt- (yeast protein transport) GTPases are homologous to mammalian Rab-GTPases, families of proteins which regulate intracellular membrane trafficking (Segev, 2001). To date, eighteen mammalian Yip family members, divided into four subfamilies, have been identified. Initially, yeast two-hybrid (Y2H) methods were used to identify an essential yeast gene termed Ypt-interacting protein 1, or Yip1p (Yang et al., 1998). Following the identification of Yip1p, several other Ypt and Yip1p-interacting proteins were identified in yeast, including Yop1p (Yip2p), Yip3p, Yip4p, Yip5p and Yif1p (Matern et al., 2000; Calero and Collins, 2002; Calero et al., 2002). Despite a low amino acid homology (<1%), all members of the yeast Yip family share an overall similar membrane topology with multiple hairpin/transmembrane domains and extended amino and/or carboxyl termini, though they appear to differ in the number of such domains and their subcellular localizations (Pfeffer and Aivazian, 2004).

The power of yeast genetic research has revealed many intriguing aspects of yeast Yip function, functions that have not completely been explored with all eighteen mammalian Yip family members. However, initial research suggested several non-related functions and structure for various yeast Yip and mammalian REEP proteins, and the discordant data suggested a possible cacophony of structure and function. But members of the mammalian Yip superfamily may have similar, yet uncharacterized, intracellular roles as their yeast counterparts, suggesting a possible harmony of functions. Prior yeast genetic research would be a reasonable starting point for hypothesis-generation surrounding mammalian Yip family members and their function. If the function of yeast Yip proteins has been maintained through evolution, then mammalian Yip family members could serve similar roles in intracellular vesicle trafficking.

The field of research on mammalian Yip proteins is confusing, in part, due to the multitude of names that have been given the various proteins based upon their original identification. Additionally, the yeast and mammalian homologs do not necessarily share the same names. For example, yeast Yip1p is homologous to mammalian Yipf5/Yip1A/FinGER5/SMAP-5 (all different published names for the same protein), further adding to the lack of clarity in the literature (Table 1). Comparisons between yeast and mammalian family members have been hampered due to different nomenclature, thus impeding interpretation of prior research. A prior review on one Yip subfamily (Yipf family) summarized these alternative names and also suggested a new nomenclature based upon structural and functional homologies of this subfamily (discussed further below) (Shaik et al., 2019). In addition, this review will discuss what is known about each mammalian Yip family, discuss unanswered questions based upon the corresponding yeast and other eukaryotic (e.g., plant and *Drosophila*) literature, as well as identify new directions for future research.

**TABLE 1:** Mammalian Yipf family.

HGNC	Alternative nomenclature (Shaik et al., 2019)	Alternative names	Yeast homolog	TM/HP domains	Localization
Yipf1 (Soonthornsit et al., 2017)	YIPFβ3A	Yip5a (Pfeffer and Aivazian, 2004)	Yip5p (Calero et al., 2002)	5	trans-Golgi (Shakoori et al., 2003; Kranjc et al., 2017; Soonthornsit et al., 2017)
		FinGER1 (Shakoori et al., 2003)			
Yipf2 (Soonthornsit et al., 2017)	YIPFβ3B	Yip5c (Pfeffer and Aivazian, 2004)	—	5	trans-Golgi (Shakoori et al., 2003; Lisauskas et al., 2012; Kranjc et al., 2017; Soonthornsit et al., 2017)
		FinGER2 (Shakoori et al., 2003)			
Yipf3 (Tanimoto et al., 2011)	YIPFβ2	Yip5b (Pfeffer and Aivazian, 2004)	—	5	cis-Golgi (Shakoori et al., 2003; Tanimoto et al., 2011; Lisauskas et al., 2012; Kranjc et al., 2017)
		FinGER3 (Shakoori et al., 2003)			
		KLIP1 (Prost et al., 2002)			
Yipf4 (Tanimoto et al., 2011)	YIPFα2	FinGER4 (Shakoori et al., 2003)	—	5	cis-Golgi (Shakoori et al., 2003; Tanimoto et al., 2011; Lisauskas et al., 2012; Kranjc et al., 2017)
Yipf5 (Yoshida et al., 2008)	YIPFα1A	Yip1a (Tang et al., 2001)	Yip1p (Yang et al., 1998)	5	cis-Golgi (Shakoori et al., 2003; Tanimoto et al., 2011; Kranjc et al., 2017)
		FinGER5 (Shakoori et al., 2003)			ER Exit Site (Tang et al., 2001; Heidtman et al., 2003)
		SMAP-5 (Stolle et al., 2005)			ERGIC (Yoshida et al., 2008; Kano et al., 2009)
Yipf6 (Brandl et al., 2012; Soonthornsit et al., 2017)	YIPFα3	Yip4 (Pfeffer and Aivazian, 2004)	Yip4p (Calero et al., 2002)	5	Golgi (Shakoori et al., 2003; Kranjc et al., 2017; Soonthornsit et al., 2017)
		FinGER6 (Shakoori et al., 2003)			
Yipf7 (Yoshida et al., 2008)	YIPFα1B	Yip1b (Tang et al., 2001)	—	5	Golgi (Tanimoto et al., 2011; Barone et al., 2015; Kranjc et al., 2017)
		FinGER9 (Stolle et al., 2005)			ERGIC (Barone et al., 2015)

In order to make this review easier to read and follow, names used for various mammalian Yip family members will conform to standard HGNC (HUGO Gene Nomenclature Committee) nomenclature, however, the literature cited may reference alternative names. By clarifying the nomenclature between different families, the cellular and biochemical similarities and differences between yeast, mammalian, and other eukaryotic homologs/orthologs can be more easily discussed. For clarity, all yeast proteins will be denoted with the standard extension “p” at the end (e.g., Yip1p), whereas mammalian proteins do not carry such a designation (e.g., Yipf5).

Prior research on the regulation of intracellular trafficking of GPCRs and neurotransmitter transporters have touched on roles for various Yip family members and intracellular transport of these proteins, however they have been written from the point of view of the cargo (Lin C.-l. G. et al., 2001; Carrel et al., 2008; Doly and Marullo, 2015). Prior individual Yip subfamily (e.g., Yipf, REEP, PRAF) reviews have touched upon similar topics (Park and Blackstone, 2010; Doly and Marullo, 2015; Shaik et al., 2019). However, this review will present an overview of all four subfamilies across multiple eukaryotic species in order to allow for comparisons between different Yip subfamilies, thus present a more complete understanding of their membrane/organelle localization, interacting proteins, and function/regulation of intracellular trafficking.

Lastly, I will discuss the concept of Yip family members as belonging to a larger superfamily of membrane-shaping adapter proteins (MSAPs), proteins that both shape membranes and act as adapters for protein-protein interactions, a novel paradigm in membrane organization (Bauer and Pelkmans, 2006). Originally, the term superfamily implied evolutionarily related proteins based upon sequence homology, however, the term has now been used in the literature to refer to a group of structurally or functionally related proteins, that may have evolved significantly so as to no longer possess high homology (Das et al., 2015). Various Yip family members have been shown to possess hairpin and amphipathic helical domains, and their biological role as membrane shaping and sensing proteins will be described. MSAPs are defined by their ability to: 1) localize to a specific membrane type(s), 2) alter membrane structure, 3) interact with other proteins via specific domains, and 4) show specificity in their interactions and effects on cargo proteins. Despite gaps, current research suggests that Yip proteins may represent the largest class of MSAPs (with eighteen members), dwarfing the next largest MSAP families of caveolin and reticulon proteins (three and four members respectively) (Bauer and Pelkmans, 2006).

### 1.1 Ypt/rab GTPases and vesicle trafficking

All cells, including neurons, have developed multiple trafficking pathways for transporting newly synthesized proteins (cargo) from the ER to Golgi to plasma membrane (secretory pathway), as well as recycling plasma membrane proteins (endocytic pathway). There appears to be multiple redundant, overlapping pathways, but in general cargo transport involves the creation of intracellular transport vesicles by fission of membrane buds, translocation down the pathway by microtubule-based motor proteins, followed by docking and fusion of vesicles and acceptor membranes (Stenmark, 2009; Pfeffer, 2017). A complete review of the protein machinery involved in intracellular membrane and cargo protein trafficking is beyond the scope of this review, however some basic concepts will be discussed. Interestingly, many neurological and other disorders appear to be caused by mutations in various trafficking proteins (De Matteis and Luini, 2011).

**FIGURE 1:**
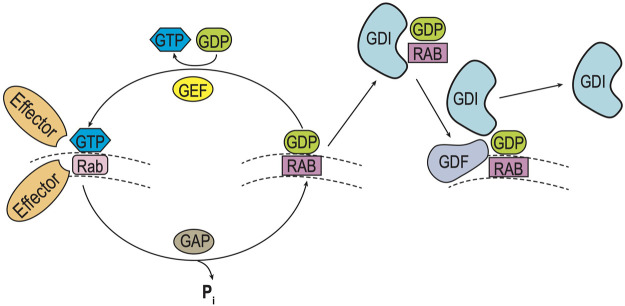
Rab GTPase Cycle. Rab (and Ypt) GTPases cycle between inactive and active forms as they move between intraorganellar membranes (e.g. ER, Golgi) (Pfeffer and Aivazian, 2004). Such membrane cycling is dependent upon the type of guanine nucleotide bound. Left: Inactive Rab-GDP is converted to its active form by exchange of GDP for GTP, catalyzed by a guanine nucleotide exchange (**GEF**) factor. Inactivation of the Rab-GTP occurs by hydrolysis of GTP, which is stimulated by a GTPase-activating protein (**GAP**). Right: Cycling of Rab-GDP between membranes requires binding of a Rab GDP dissociation inhibitor (**GDI**), to prevent Rab activation as it moves through the cytoplasm. Ultimately specific membrane targeting is mediated by a membrane-bound GDI displacement factor (**GDF**), which releases Rab-GDP from the GDI to allow for insertion into its cognate membrane.

Movement of higher eukaryotic protein cargo is dependent upon a family of proteins termed Rab-GTPases (Rabs), homologous to yeast Ypt-GTPases (Ypts) (Lipatova et al., 2015). These small GTPases are localized to cytoplasmic surfaces of various organelle membranes, where they assist in transport vesicle formation, linkage to motor proteins, and ultimately docking and fusing of vesicles with target membranes (Pfeffer and Aivazian, 2004; Stenmark, 2009). Ypts/Rabs serve as scaffolds to recruit effector proteins (e.g., motor proteins, SNAREs, tethers) to various membrane compartments (e.g., ER, Golgi, endosomes), in order to regulate intracellular traffic (Figure 1).

Ypt/Rabs cycle between GDP-bound (inactive) and GTP-bound (active) states, termed Ypt/Rab-GDP and Ypt/Rab-GTP respectively. This cycle is controlled by a guanine nucleotide exchange factor (**GEF**) which triggers the displacement of GDP by GTP to activate Ypt/Rabs and GTPase-activating protein (**GAP**) which enhance hydrolysis of GTP to GDP to inactivate Ypt/Rabs. In addition, Ypts/Rabs are prenylated to allow for membrane attachment and cycling between organelle compartments. Following inactivation by GAP, membrane-bound Rab-GDPs are extracted by a GDP-dissociation inhibitor (**GDI**) which allows the prenylated Ypt/Rab-GDP to remain cytosolic and inactive until it returns to its cognate membrane. Final recruitment and localization of specific Ypt/Rab-GDP proteins back to its cognate membrane requires a GDI-displacement factor (**GDF**) to release the prenylated Ypt/Rab-GDP from the GDI, and allow for membrane attachment (Pfeffer and Aivazian, 2004; Grosshans et al., 2006; Stenmark, 2009).

### 1.2 Identification of yeast ypt-interacting proteins (yip family)

With over sixty mammalian Rabs and eleven yeast Ypts, it appears that a high level of specialization has evolved to regulate each of these trafficking steps between organelles (Stenmark, 2009). Due to its relative simplicity, yeast has been utilized as a model system for neuronal secretion and multiple components of the molecular machinery mentioned above have been delineated in both systems (Bennett and Scheller, 1993). Initially, Y2H methods were used to identify an essential yeast gene termed Ypt-interacting protein 1, or Yip1p. This integral Golgi membrane protein was shown to interact directly with several, but not all Ypts tested, suggesting specificity of function (Yang et al., 1998). Depletion of Yip1p led to ER membrane accumulation and aberrant protein secretion and glycosylation (Yang et al., 1998).

Following the characterization of Yip1p, several other Ypt and Yip1p-interacting proteins were discovered. Yif1p was identified as an integral membrane protein that tightly bound Yip1p on Golgi membranes (Matern et al., 2000). In addition, the amino terminus was shown to be cytosolic and bound Ypts. Loss of Yif1p function led to a block of ER-Golgi transport and an accumulation of ER membranes and vesicles. Soon after the discovery of Yip1p and Yif1p, another family member was discovered, Yop1p (also termed Yip2p) (Calero et al., 2001). Similar to the other proteins, Yop1p and Yip1p interacted through their cytosolic amino termini and bound Ypts. Yop1p was not an essential gene, however overexpression of Yop1p had unique cellular effects. Overexpression led to an accumulation of internal ER membranes and a block in membrane trafficking, again leading to aberrant core glycosylation of model secreted proteins. Three more yeast proteins were identified soon after, each with a similar membrane topology of presumed transmembrane/hairpin domains flanked by cytosolic amino and carboxy termini. The first, Yip3p, was again shown to interact with Ypt1p and Ypt31p, as well as Yip1p. More importantly, it was demonstrated that Yip3p only bound prenylated Ypts and even some mammalian Rabs (Calero and Collins, 2002). Lastly, Yip4p and Yip5p were identified as other members of this yeast Yip1 family (Calero et al., 2002). These proteins were identified by functional cloning, and despite having similar cellular functions and predicted topologies, they share <1% amino acid identity (data not shown).

Given the biochemical evidence of Yip family/Ypt interactions, is there evidence that they are incorporated into intracellular vesicles, further supporting their role in vesicle trafficking? As briefly mentioned above, several independent studies have supported this hypothesis. First, Yip1p was shown to be involved with early-stage ER/COPII vesicle budding, and it was hypothesized the vesicle biogenesis was coupled to cargo loading of vesicles (Heidtman et al., 2003). Second, the Yip1p/Yif1p complex was required to make ER-derived vesicles “fusion competent” and this complex bound SNAREs (e.g., Bos1 and Sec22 proteins) necessary for ER/Golgi fusion (Barrowman et al., 2003). Third, Yip1p, Yip3p, and Yif1p were identified in purified COPII vesicles, and it was shown that they were efficiently packaged into these vesicles (Otte et al., 2001). The remaining family member, Yop1p, was not yet cloned at the time of these latter studies so similar evidence for its possible role in vesicle biogenesis/trafficking was not examined.

Currently, eighteen mammalian homologs of the yeast Yip superfamily have been identified, leading to a re-classification into four separate subfamilies: Yipf, REEP (Yip2), Yif1, and PRAF (PRA = Prenylated Rab Acceptor, Yip3) families (Supplemental Figure S1, Supplemental Table S1) (Pfeffer and Aivazian, 2004). Because many of these proteins were discovered by different laboratories, they have multiple names, which will be clarified in subsequent sections of this review. Additionally, published research on mammalian Yip2/Yip3 families have not formally adopted the “Yip” nomenclature and these families will be referred to as REEP/PRAF families respectively.

## 2 Yeast Yip/Mammalian Yipf family

### 2.1 Yeast Yip family

**FIGURE 2:**
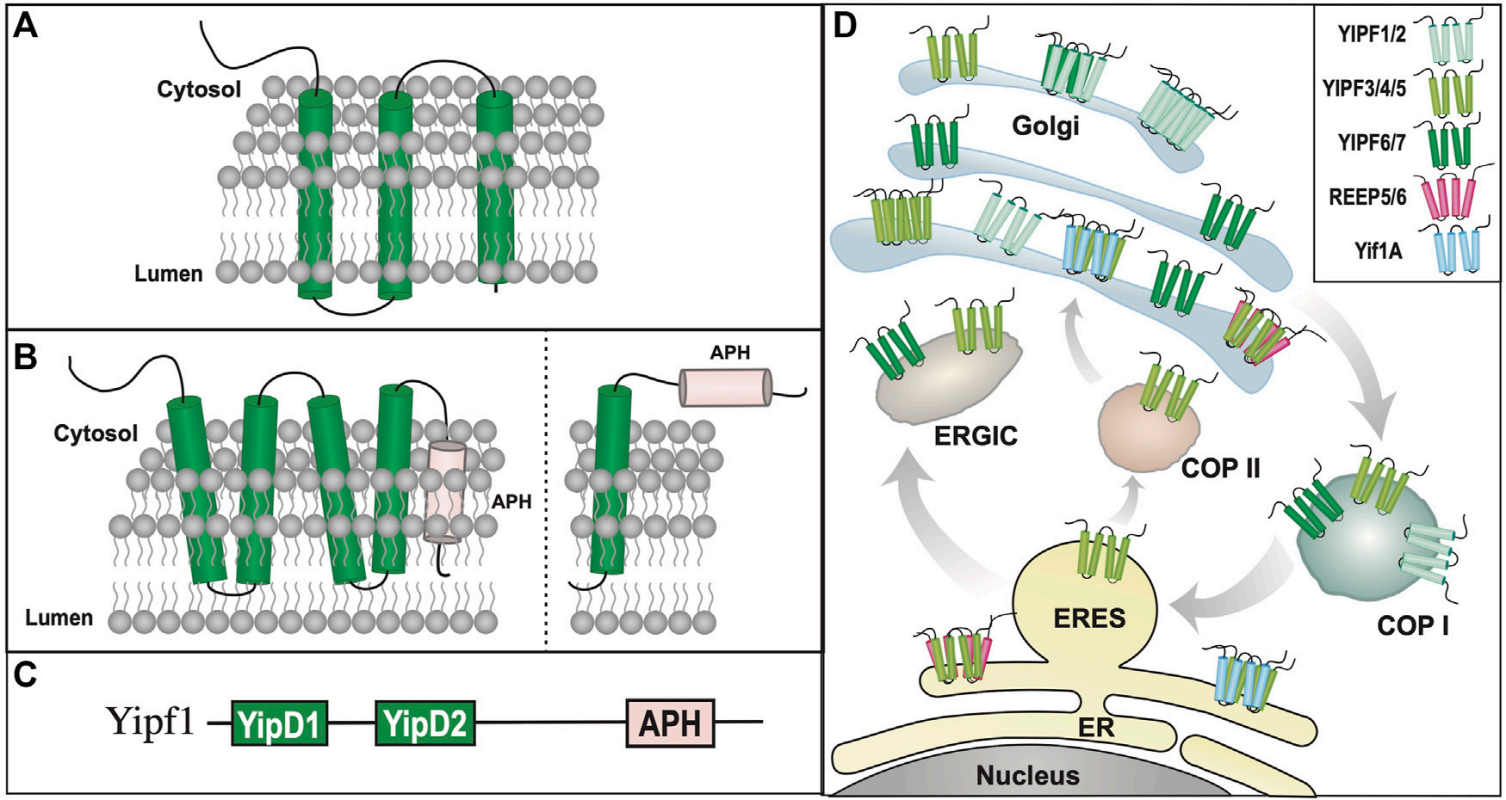
Yipf/Yip1p Family of Proteins. **(A).** When originally discovered, Yip1p was modeled as a three transmembrane domain containing protein (Yang et al., 1998). **(B). Left:** Alternative transmembrane topology model of Yip4p and Yip5p based on further biochemical analyses (Calero et al., 2002). Note that the membrane localization of the carboxy terminus of Yip4p/5p was determined to be intraluminal. **Right:** An alternative model predicted by AlphaFold (Jumper et al., 2021; Varadi et al., 2022), and yeast Yip4p/5p data (Calero et al., 2002), suggesting that their carboxy termini are closely aligned within the ER/Golgi membrane, possibly as an APH that is either aligned or buried within the membrane. **(C).** Yip family members have two conserved Yip1 Domains (**YIPD**) that may insert into the membrane as dual hairpin structures, which are necessary for Yip family homomeric and heteromeric interactions. Additionally, all members possess a carboxy terminal amphipathic helical domain (**APH**). **(D).** Intracellular localization of various Yipf family members is shown within the ER, Golgi, and ERGIC compartments, including intracellular transport vesicles, COPI and COPII. Modeled proteins are not shown to scale relative to their amino acid sequence. ER = Endoplasmic Reticulum, ERES = ER Exit Site, ERGIC = ER/Golgi Intermediate Compartment.

As discussed above, initial identification and characterization of yeast Yip1p and Yif1p demonstrated that these yeast proteins were essential gene products, localized to the Golgi apparatus, and that loss of function mutations led to a block of ER to Golgi vesicle transport and accumulation of ER membrane (Yang et al., 1998; Matern et al., 2000). Yip1p was originally cloned by Y2H screening for proteins that bound Ypt1p and Ypt31p and their physical interactions were further confirmed by affinity chromatography and co-immunoprecipitation assays (Yang et al., 1998). Cellular studies using either lethal Yip1p mutants or Yip1p-depleted yeast revealed a massive accumulation of ER membranes, accompanied by aberrant protein glycosylation and secretion. Lastly, subcellular fractionation and indirect immunofluorescent assays demonstrated that Yip1p was localized to Golgi membranes at steady state. The structure and intracellular membrane orientation of Yip1p was not delineated beyond identification of “three” putative transmembrane domains (Figure 2A). Similar cellular mutation/deletion studies have not been performed with Yip4p and Yip5p, however they were subsequently placed within the original Yip1p family, whereas Yop1p (Yip2p) and Yip3p were placed into separate yeast Yip protein subfamilies.

#### 2.1.1 Yeast Yip cell biology/biochemistry

Initial *in vitro* biochemical analysis of Yip1p-Ypt interactions suggested a lack of Ypt specificity for Yip1p binding. Additionally it was demonstrated that Yip1p could only bind di-prenylated, not mono-prenylated, Ypt proteins and lastly, that the human ortholog (Yipf5) could fully replace loss of Yip1p within yeast (Calero et al., 2003). Further analysis of Yip1p-binding specificity for Ypt proteins was undertaken, which demonstrated that specific Ypt isoforms required for Yip1p function were localized to Golgi membranes and that Yip1p mutations that negatively impacted ER vesicle budding also did not interact with Ypt proteins (Chen et al., 2004). Using a cell-free assay, it was further determined that Yip1p can cycle between the ER and Golgi, though it again appeared that it was preferentially localized to the Golgi apparatus at steady-state (Yang et al., 1998). Additionally it was shown that Yip1p was a constituent of ER-derived transport vesicles, and that Yip1p function was necessary for biogenesis of COPII-derived vesicles (Otte et al., 2001; Heidtman et al., 2003). Other laboratories reported similar findings, that a Yip1p-Yif1p complex was required to produce ER-derived fusion competent vesicles for fusion with Golgi membranes (Barrowman et al., 2003). Yip4p and Yip5p were subsequently identified, based upon their interactions with Yip1p and Yif1p (Table 1); their gene products were determined to be non-essential to yeast (Calero et al., 2002). Similar to Yip1p, Yip4p and Yip5p also bound specific prenylated Ypts, as well as another Rab GTPase termed Sec4p, which is involved with the final yeast exocytic secretory pathway from Golgi to plasma membrane (Table 2).

**TABLE 2:** Yipf family interacting proteins.

Mammalian	Mammalian interacting proteins	Yeast	Yeast interacting proteins
Yipf1	Yipf6 (Shakoori et al., 2003)	Yip5p	Ypt1/7/10/11/31/32/52/53p (Calero et al., 2002)
	Yipf2 (Shakoori et al., 2003)		Yip4p (Calero et al., 2002)
			Sec4p (Calero et al., 2002)
Yipf2	Yipf6 (Shakoori et al., 2003)	—	—
	Yipf1 (Shakoori et al., 2003)		
	Rab5, Rab22a (Qi et al., 2019)		
	Rab8 (Wang et al., 2020)		
	CD147 (Qi et al., 2019)		
	TNFRF10B (Wang et al., 2020)		
Yipf3	Yipf4 (Tanimoto et al., 2011)	—	—
Yipf4	Yipf3 (Tanimoto et al., 2011)	—	—
Yipf5	—	Yip1p	Ypt1/31p (Yang et al., 1998; Matern et al., 2000; Chen et al., 2004)
	—		Yip4p (Calero et al., 2002)
	Yif1A (Jin et al., 2005)		Yif1a (Matern et al., 2000)
	Sec23/24 (COPII) (Tang et al., 2001)		Yop1p (Calero et al., 2001)
	REEP5 (Dykstra et al., 2010)		Yos1p (Heidtman et al., 2005)
	—		Bos1p/Sec22p (Barrowman et al., 2003)
	—		COPII vesicles (Otte et al., 2001; Heidtman et al., 2003)
	—		SNC2p/TLG1p (Ito et al., 2001)
Yipf6	—	Yip4p	Ypt1/6/7/10/11/31/32/52/53p (Calero et al., 2002)
	Yipf1 (Shakoori et al., 2003)		Yip1p (Calero et al., 2002)
	Yipf2 (Shakoori et al., 2003)		Yip5p (Calero et al., 2002)
	—		Sec4p (Calero et al., 2002)
Yipf7	—	—	—

Other Yip1p-interacting proteins, beyond Ypts have been described, including multiple SNARE proteins (Bos1p, Sec22p, and vSNARE/SNC2) involved with intracellular membrane transport (Table 2) (Ito et al., 2001; Barrowman et al., 2003). In addition, a novel yeast integral membrane protein Yos1p (Yip One Suppressor 1) was identified as another Yip1p binding partner (Heidtman et al., 2005). Yos1p was localized to the ER and Golgi, where it was demonstrated to be efficiently packaged into COPII vesicles, and its deletion blocked ER to Golgi trafficking. However, it is not known how Yip1p binding to these various proteins alter their function or vice versa. Lastly, it appears that Yip1p can form complexes with other members of the larger yeast Yip family, including Yif1p (Matern et al., 2000), Yip4p (Calero et al., 2002), and Yop1p (Calero et al., 2001), but very weakly with Yip5p (Calero et al., 2002). To date, no specific cargo protein interactions have been identified for any yeast or mammalian Yip family members, as will be discussed further for other members of the larger mammalian Yip family (e.g., REEP, Yif, and PRAF families).

#### 2.1.2 Yeast Yip structure/topology

Protease analysis of isolated membranes strongly suggested that the amino terminal portion of Yip1p was cytosolic, and not intraluminal within Golgi or ER membranes, and it was modeled with three transmembrane domains (Yang et al., 1998) (Figure 2A). Furthermore, it was shown that the cytosolic amino terminus of Yip1p interacted with the hydrophilic amino terminus of Yop1p. Within a cell-free assay system, addition of an antibodies directed against the amino-terminal domain of Yip1p inhibited budding of COPII-derived vesicles, suggesting a role for the amino terminus of Yip1p in its function (Heidtman et al., 2003). However, deletion of the amino-terminal 65 amino acids of Yip1p did not alter the growth profile when expressed in yeast, whereas deletion of 18 amino acids from the carboxy terminus resulted in mutant protein that could not complement Yip1p deletion (Chen et al., 2004). Specific protein domains or functions within these deleted regions were not identified. Therefore, little is known about the structure or specific domains of yeast Yip family members compared to other mammalian Yip subfamilies.

Despite the low sequence homology between Yip1p, Yip4p, and Yip5p, they were modeled with different topologies. Unlike Yip1p, Yip4p and Yip5p were modeled as possessing five transmembrane/alpha-helical domains, instead of three transmembrane domains. Based upon their homology to Yip1p, it was suggested that both Yip4p and Yip5p possess a cytosolic facing amino terminus, however unlike Yip1p, initial topological analysis predicted that the carboxy terminal “transmembrane” domains of Yip4p and Yip5p were not cytosolic, but largely buried in the membrane. (Figure 2B) (Calero et al., 2002). The identification of Yip4p and Yip5p led to the concept of a larger Yip1p family of proteins (including Yip1p, Yif1p, Yop1p, and Yip3p) that shared the following features:

1) they have a common domain topology, specifically multiple transmembrane/hairpin domains (termed the “Yip1 domain” = YipD) flanked by cytosolic amino and membrane inserted carboxy termini
2) they interact with Ypts/Rabs
3) they associate with other members of the Yip1p family and intracellular transport machinery proteins

### 2.2 Mammalian Yipf family

Following the identification of Yip1p, two mammalian family members most closely related to Yip1p were identified from an EST database, Yip1A (Yipf5) and Yip1B (Yipf7) (Tang et al., 2001). Subsequently, Shakoori and colleagues identified eight related mammalian family members based upon a protein database search with Yip1p, Yif1p, Yip4p and Yip5p. They named the newly identified proteins “Five-pass transmembrane proteins localizing in the Golgi apparatus and in the ER” or “FinGER1-8 (Shakoori et al., 2003). The eight proteins could be separated into two subfamilies based on sequence homology, FinGER1-6 and FinGER7-8, and an additionally family member (FinGER9) was identified shortly thereafter (Stolle et al., 2005). FinGER1-6/9 belong to one Yip family that shares the most homology with Yip1p and they have been renamed Yipf1-7 respectively (Table 1).

As was seen with Yip1p-Yip5p, the homology amongst Yipf1-7 is low, approximately 10% (Supplemental Table S1, Supplemental Figure S3). FinGER7/8 shared the most homology with Yif1p and thus were separated into the Yif1 subfamily, which will be discussed further in a subsequent section. To avoid confusion and to use a common set of nomenclature, the first mammalian Yip family was renamed the “Yip1 Domain Family” or Yipf (Pfeffer and Aivazian, 2004). The Yipf family was named based on the conserved “Yip1 Domain” (YipD), a conserved transmembrane region of Yip1p, Yip4p, and Yip5p found in all nine FinGER proteins (Figure 2C).

As mentioned earlier, the yeast and mammalian names do not directly correlate (i.e., Yip4p is not homologous to Yipf4), but based upon sequence homology, it was determined that Yipf1 was most homologous to Yip5p, Yipf5 was most closely related to Yip1p, and Yipf6 was similar to Yip4p (Table 1). Additionally, several other research groups identified various members of the mammalian Yipf family based upon their function, leading to further alternative names distinct from the Yip nomenclature (e.g., KLIP1, SMAP-5) (Prost et al., 2002; Stolle et al., 2005). Yipf1-3 are most similar to Yip5p and hence were alternatively named Yip5a-c (Pfeffer and Aivazian, 2004). It is apparent that higher eukaryotes have evolved to require more Yipf family members than the three originally discovered in yeast.

#### 2.2.1 Mammalian Yipf cell biology/biochemistry

Initial characterization of Yipf5 and Yipf7 revealed several similar cellular and biochemical functions as Yip1p (Tang et al., 2001). For example, sequence analysis suggested a similar transmembrane and amino terminal structure between the yeast and mammalian orthologs. Additionally, both Yipf5 and Yipf7 demonstrated similar localization of ER exit sites (ERES), where COPII vesicle biogenesis originates. Over-expression of the amino terminus of Yipf5 led to disruption of the Golgi apparatus implicating Yipf5 in regulation of ER-Golgi transport. Lastly, it was demonstrated that amino terminal region of Yipf5 bound two proteins that form a subcomplex of the COPII coat, specifically Sec23/Sec24, similar to Yip1p interactions with Bos1p/Sec22p (Table 2). Preliminary unpublished data suggested that the highly conserved transmembrane region of Yipf5 (“Yip1 Domain”—amino acids 75–106) was important for interaction with Sec23 (Figure 2C) (Tang et al., 2001). Though a role for Yip1p and Yipf family members in COPII-mediated vesicle transport had been established, it was also demonstrated that Yipf5 regulated COPI-independent retrograde transport from the Golgi complex to the ER (Kano et al., 2009).

When compared to yeast Yip1 family members, the newly described mammalian Yipf family appeared to have similar functions in ER COPII vesicle biogenesis, transmembrane structure, subcellular localization, and interacting proteins. Yipf5 was shown to interact with COPII vesicle proteins Sec23/24, similar to Yip1p (Tang et al., 2001), however, interactions with specific Rabs were not delineated, except demonstration that Yipf5 did not bind Rab1. However, knockdown of Yipf5 led to the release of a single Rab isoform, Rab6, from Golgi membranes, the first example of a role for Yipf family members and membrane recruitment of a Rab protein (Kano et al., 2009).

Rab isoform release from, and insertion into, a cognate organelle membrane requires a Rab-GDI or Rab-GDF respectively, suggesting a possible role for Yipf family members in Rab cycling (Figure 1). Interestingly, using yeast genetic analyses, it was demonstrated that Yip1p function *in vivo* intersected with Rab-GDI activity suggesting a common pathway to affect Rab function (Chen et al., 2004). Recently, cell biological experiments demonstrated that Yipf2 could interact with specific Rab proteins, potentially as a Rab-GDF, leading to alterations in Rab membrane recruitment and activation (Qi et al., 2019; Wang et al., 2020). First, using co-immunoprecipitation assays, it was shown that Yipf2 could interact directly with all Rab5, Rab22a, and Rab8. Second, when Yipf2 expression was knocked-down, ER localization of Rab5 and Golgi localization of Rab22a were reduced specifically in these organelles.

Initial subcellular localization studies were performed on Yipf1-7, utilizing FLAG- and HA-epitope tagged constructs, demonstrating some differences between the various family members (Shakoori et al., 2003). Depending upon the level of heterologous expression of epitope-tagged constructs, it was shown that Yipf3/Yipf4 were localized to the cis-Golgi, whereas Yipf5 and Yipf7 were localized to ER as shown previously (Tang et al., 2001). High expression levels of these epitope-tagged constructs revealed that all Yipf family members could also be found in the ER, however the authors acknowledged that this may reflect accumulation of over-expressed proteins and not their native localization.

To overcome the limitations of heterologous expression of epitope-tagged proteins, similar experiments were undertaken with specific Yipf antisera. As suggested by prior research, it was demonstrated that Yipf1, Yipf2, and Yipf6 localized to the Golgi apparatus, both by immunofluorescent staining and membrane fractionation experiments with Golgi marker proteins (Figure 2D) (Soonthornsit et al., 2017). Yoshida and colleagues analyzed endogenous Yipf5 localization and function using immunofluorescent staining, subcellular fractionation, and immunoprecipitation experiments with specific Yipf5 antisera (Yoshida et al., 2008). Their research demonstrated that Yipf5 was localized to ER, ERGIC (ER Golgi Intermediate Compartment), and cis-Golgi membranes at steady-state, most likely reflecting recycling between the different membranes, and was confirmed by others (Kano et al., 2009). Similarly, it was demonstrated that endogenous Yipf7 protein was localized to the cis-Golgi and ERGIC compartments (Barone et al., 2015). Tanimoto and colleagues further characterized endogenous Yipf3 and Yipf4 localization with specific antisera raised against the proteins, demonstrating that they too are localized to the cis-Golgi (Tanimoto et al., 2011). Additionally, membrane fractionation experiments demonstrated co-localization with Golgi marker proteins. However, they also demonstrated that Yipf3 underwent glycolytic processing, and that the various forms localized to different compartments. For example, the initial N-glycosylated form of Yipf3 was found in the ER or ERGIC, and as it underwent glycolytic processing, moved to the Golgi. Recently, a more complete analysis of all seven Yipf family members was undertaken with GFP constructs, revealing that various members localize to different specific Golgi regions (Kranjc et al., 2017). It was demonstrated that Yipf1/2 localized predominantly to trans-Golgi membranes, whereas Yipf3/4/5 localized to the cis-Golgi. However, Yipf6/7 localized through both the cis- and trans-Golgi membranes.

Whereas deletion of Yip1p was lethal to yeast (Yang et al., 1998), knockdown experiments with mammalian Yipf family members did not demonstrate such lethality, but it did lead to alterations in intracellular structures. For example, knockdown of Yipf5 led to partial disassembly of the Golgi apparatus and it was shown that knockdown of either Yipf3 or Yipf4 led to fragmentation of the Golgi apparatus, suggesting a possible role in maintaining Golgi structure (Yoshida et al., 2008). Further analysis of Yipf5 depletion, demonstrated that loss of this protein led to restructuring of the ER membrane into ‘whorls’ and membrane stacking was observed, coincident with a marked slowing of COPII-mediated protein export (Dykstra et al., 2010). These observations were not seen when ER export was blocked biochemically. Further mutational analysis of Yipf5 structure showed that two highly conserved amino acids within the cytosolic amino terminal (E^95^) and transmembrane regions (K^146^) (Yip1p E^76^ and K^130^ respectively), where necessary for whorl formation (Dykstra et al., 2013). Interestingly, the two corresponding yeast Yip1p amino acids were shown to be essential for yeast viability (Chen et al., 2004). Though membrane-shaping by Yipf5 was not demonstrated, it appears that Yipf5 does impact intracellular organelle shape and organization.

Much like members of the yeast Yip1 family, Yipf family members demonstrate protein-protein interactions (Table 2). Yipf1, Yipf2 and Yipf6 were shown to interact strongly with each other, but only weakly with Yipf3, Yipf4, or Yipf5 (Shakoori et al., 2003; Soonthornsit et al., 2017). Other laboratories demonstrated that Yipf3 andYipf4 interact within the Golgi (Tanimoto et al., 2011). Additionally, interactions between Yipf5 and Yif1 were demonstrated, similar to that described previously for their yeast orthologs, Yip1p and Yif1p respectively (Yoshida et al., 2008). Initially analysis suggested that the interaction between Yipf1 and Yipf6 required the complete transmembrane (Yip1 domain) region; the amino or carboxy terminal regions were not necessary for their interaction. Lastly, analogous to Yip1p and Yop1p interactions, it was demonstrated that Yipf5 did interact with REEP5, a mammalian Yop1p ortholog (Dykstra et al., 2010).

#### 2.2.2 Mammalian Yipf structure/topology

Overall, sequence comparisons between the yeast and mammalian Yip1/Yipf families revealed three highly conserved regions, including an amino terminal hydrophilic domain near the first transmembrane domain (Yip1 Domain), as well as unique transmembrane domains that contained either a conserved proline or glycine residues (Shakoori et al., 2003). Further specifics about conserved amino acid motifs found in Yipf family members has been reviewed previously (Shaik et al., 2019). Initial analysis suggested five transmembrane domains; however, the possibility of hairpin structures seen in other Yip family members was not discussed (Voeltz et al., 2006). Similarly, biochemical analysis of Yipf1-3, revealed that the amino terminus was exposed to the cytoplasm, whereas the carboxy terminus was either intraluminal or extracellular.

Recently, the membrane topology of all seven members of the Yipf family was determined utilizing a fluorescence protease protection (FPP) assay (Kranjc et al., 2017). It was modeled upon five alpha helical domains, with the first four helical domains found within the membrane, and a fifth alpha helical domain that appeared to not traverse the membrane. There experimental studies suggested that the amino terminus was cytosolic, whereas the carboxy terminus was intraluminal, residing within Golgi membranes (Figure 2B). Other work predicted that Yip4p and Yip5p had two transmembrane domains (possible hairpins?) and a carboxy terminal domain buried, but not inserted into, the membrane (Calero et al., 2002). Recently, a highly accurate artificial intelligence system (AlphaFold) was developed to predict a protein’s 3D structure based upon its amino acid sequence (Jumper et al., 2021; Varadi et al., 2022). Using this system, Yipf members are predicted to have four transmembrane domains with the fifth carboxy alpha-helical domain being cytosolic, possibly as an APH domain (Supplemental Figure S3). Based upon the topology and homology of Yipf proteins with other Yip subfamily members (see below), it is tempting to speculate that the fifth transmembrane domain may in fact function as an APH domain aligned with, but not traversing, the membrane, but this remains to be verified experimentally (Figure 2B). Therefore, current experimental data demonstrates that mammalian Yipf family members have five transmembrane domains, with a cytosolic amino terminus and luminal carboxy terminus, whereas other experimental evidence suggests that yeast Yip4p and Yip5p have a carboxy terminal domain buried in the membrane.

### 2.3 Yip/Yipf family summary

Overall, a growing body of research suggests that many of the cellular and biochemical functions and properties of Yip1p, Yip4p, and Yip5p have been conserved in the mammalian Yipf family. For example, members of this family are localized to ER and Golgi membranes, including the ERGIC, and appear to be involved with transport of COPII vesicles between these two membranous structures, similar to Yip1p. However, it appears that mammalian Yipf family has evolved to include more members (seven) than that found in yeast (three), possibly due to the increased complexity of mammalian intracellular transport. In addition to subcellular localization, knockdown or knockout of various Yip1p or Yipf gene products led to similar changes in ER and Golgi membrane structure suggesting a role for these proteins in maintenance of these organelles. Similar to that seen with Yip1p/Yip4p/Yip5p, protein-protein interactions between different Yipf family members have been determined. It also was demonstrated that Yipf5 bound components of COPII machinery, specifically Sec23/24 proteins (Tang et al., 2001), analogous to Yip1p interactions with Bos1p/Sec22p or Yip4p/Yip5p with Sec4p (Calero et al., 2002; Barrowman et al., 2003). Therefore, it appears that many of the intracellular transport functions of Yip1p and its yeast relatives have been conserved in the Yipf family (Figure 2D).

Compared to the other Yip subfamilies, the Yipf subfamily has the most confusion surrounding nomenclature. The HGCN nomenclature was based in part upon the original cloning of FinGER1-9, not based upon sequence similarity or function (Shakoori et al., 2003). Shaik and co-authors have proposed an alternative nomenclature (Table 1), combining the Yipf and Yif subfamilies, and renaming them based upon shared characteristics such as distinct complex formation and organelle localization (Shaik et al., 2019). In this nomenclature, Yip1p homologs are termed Yipfα and Yif1p homologs are named Yipfβ, where three distinct complexes are formed by special pairs of Yipfα and Yipfβ, numbered according to their Golgi localization. Specifically, the early Golgi/ERGIC resident Complex 1 consists of YIPFα1 and YIPFβ1, the middle Golgi (*cis*- Golgi) resident Complex 2 comprises YIPFα2 and YIPFβ2, and the late Golgi (*medial*-/*trans*-Golgi/TGN) Complex 3 contains YIPFα3 and YIPFβ3. Though not as widely accepted as the HGCN nomenclature, this naming system considers both function and homology.

Though Yip1p was identified by its ability to bind Ypt proteins, and further analysis demonstrated specific Ypt interactions with Yip1p, Yip4p, and Yip5p (Table 2), only a few specific Rab interactions (Yipf2 and Rab5, Rab22a, Rab8) have been identified for Yipf family members. A specific role for Yipf2 in Rab cycling as a GDF has been proposed based upon indirect cell biological experimentation, however, more complete biochemical evidence supporting the role of any Yipf family members as a Rab-GDF (as discussed for Yip3p/PRAF below) remains to be shown. *In silico* analysis of genetic databases has suggested specificity of Rab interactions with various Yipf proteins, however, these results have not been confirmed by biochemical or cellular methods and thus were not included in this review (Gurkan et al., 2005). However, yeast genetic analysis suggested an intersection between Yip1p and Rab-GDI signaling, alluding to a role for Yipf and Rab function (Chen et al., 2004). Given the overall similarity in cellular, biochemical, and structural properties between yeast and mammalian orthologs of Yipf, it is more likely that such specific Rab interactions will be identified.

One function of other mammalian Yip family members (see REEP, Yif, and PRAF families below) is their role as adapter proteins for specific cargo protein transport. In the case of REEP, Yif, and PRAF family members, specific cargo proteins have been identified whose trafficking through the ER to Golgi to plasma membrane are regulated in part by these family of proteins (Björk et al., 2013). However, few cargo proteins have yet been identified for mammalian Yipf members, nor for their yeast counterparts. Yipf2 appears to regulate plasma membrane expression and endocytic cycling of CD147 and TNFRF10B, though specific details about the regulatory mechanisms of Yipf domains involved remain to be determined (Qi et al., 2019; Wang et al., 2020).

As for transmembrane structure, the originally cloning of the Yipf family (FinGER proteins) suggested five transmembrane domains (Shakoori et al., 2003), similar to their yeast counterparts. However, as will be discussed later in this review, other members of the larger Yip family have been shown to have hairpin structures, not traditional transmembrane-spanning helices, as well as APH domains that align with the membrane and are important for protein function (Voeltz et al., 2006; Park et al., 2010). Yop1p and REEPs have been shown to insert into and alter the membrane via hairpin and amphipathic helical structures, but such membrane shaping properties have not been demonstrated yet for Yipf family members. The conserved ‘Yip1 domain’ or YipD identified in all Yipf family members is homologous in position to the REEP/Yop1p hairpin domains (RHD), where it is termed the ‘Reticulon Homology Domain’ or RHD (Voeltz et al., 2006). Lastly, binding of other adapter or structural proteins (e.g., 14-3-3 family members, tubulin) has not been investigated, hence Yip/Yipf family members do not yet fulfill the requirements to be classified as membrane-shaping adapter proteins.

## 3 Yeast Yop1p/Mammalian REEP family

### 3.1 Yeast Yop1p

Yop1p (Yip One Partner 1) was originally identified by Y2H screening for Yip1p interacting proteins (Table 3) (Calero et al., 2001). The “bait” utilized in the screen was the cytoplasmic amino terminus of Yip1p, which was subsequently shown to be the major site of interaction between these two proteins. In an analogous fashion, the first seventeen amino acids of the presumed cytoplasmic amino terminus of Yop1p were shown to be necessary for Yip1p interactions. Initially subcellular localization studies using overexpressed HA-tagged Yop1p demonstrated expression within the Golgi and possibly the ER, similar to Yip1p. Yop1p appeared to have two transmembrane domains, unlike Yip1p which appeared to have five transmembrane domains, though the exact topology was not settled.

**TABLE 3:** Mammalian REEP/Yip2 family.

HGNC	Alternative names	Yeast homolog	TM/HP domains	Localization	Cargo
REEP1	Yip2a (Pfeffer and Aivazian, 2004)	—	2	ER (Voeltz et al., 2006; Park et al., 2010; Björk et al., 2013)	ORs (Saito et al., 2004)
	ReepA (Napoli et al., 2019) (*Drosophila*)				TAS2R (Behrens et al., 2006)
	—				α2_C_AR (Björk et al., 2013)
REEP2	Yip2d (Pfeffer and Aivazian, 2004)	—	2	ER (Björk et al., 2013)	ORs (Saito et al., 2004)
					T1R2/T1R3 (Ilegems et al., 2010)
					α2_C_AR (Björk et al., 2013)
REEP3	Yip2b (Pfeffer and Aivazian, 2004)	—	2	—	—
REEP4	Yip2c (Pfeffer and Aivazian, 2004)	—	2	—	—
REEP5	Yip2e (Pfeffer and Aivazian, 2004)	Yop1p (Calero et al., 2001)	2	ER (Björk et al., 2013)	—
	TB2 (Kinzler et al., 1991; Nishisho et al., 1991)				
	DP1 (Joslyn et al., 1991)				
REEP6	Yip2f (Pfeffer and Aivazian, 2004)	—	2	—	α2_C_AR (Björk et al., 2013)
	TB1 (Nishisho et al., 1991; Sato et al., 2005)				
	DP1L1 (Sato et al., 2005)				

#### 3.1.1 Yop1p cell biology

Unlike the lethality of Yip1p deletion (Yang et al., 1998), loss of Yop1p was not fatal (Calero et al., 2001). In fact, overexpression of either full-length or the carboxy terminus of Yop1p demonstrated a dominant negative phenotype, with expression of either form leading to swollen cells of aberrant shape. In addition, overexpression of full-length Yop1p led to distortion of Golgi structures, and transport of a model protein (vacuolar protease CPY) was blocked at the level of ER-Golgi traffic, resulting in accumulation of an ER core-glycosylated form of CPY. Interesting, overexpression of Yip1p could overcome the block induced by Yop1p overexpression and reverse the subcellular structural effects seen. Lastly, it was demonstrated that similar to Yip1p, Yop1p did interact specifically with a subset of Ypt proteins (e.g. Ypt6/7/52p), as well as the SNARE protein Sec4p, which also was shown to interact with Yip4p and Yip5p (Table 4) (Calero et al., 2002).

**TABLE 4:** REEP/Yip2 family interacting proteins.

Mammalian	Mammalian interacting proteins	Yeast	Yeast interacting proteins
REEP1	Atlastin 1–3 (Hu et al., 2009; Park et al., 2010)	—	—
	M1-Spastin (Park et al., 2010)		
	Protrudin (Hashimoto et al., 2014)		
	Tubulin (Park et al., 2010)		
	14-3-3 Protein Family (Tinti et al., 2012)		
	Seipin (Renvoise et al., 2016)		
	Sey1 (Hu et al., 2009) (*Arabidopsis*)		
REEP2	Rab1b/3a (Gurkan et al., 2005)	—	—
	Tubulin (Park et al., 2010)		
	14-3-3 Protein Family (Tinti et al., 2012)		
REEP3	Tubulin (Park et al., 2010)	—	—
	14-3-3 Protein Family (Tinti et al., 2012)		
REEP4	Rab1b/3a (Gurkan et al., 2005)	—	—
	RAB3GAP-1/2 (Tinti et al., 2012)		
	Tubulin (Park et al., 2010)		
	14-3-3 Protein Family (Tinti et al., 2012)		
REEP5		Yop1p	Ypt6/7/52p (Calero et al., 2001)
	Yipf5 (Dykstra et al., 2010)		Yip1p (Calero et al., 2001)
	Protrudin (Chang et al., 2013; Hashimoto et al., 2014)		Yif1p (Heidtman et al., 2003; Shibata et al., 2008)
	Rtn3c (Shibata et al., 2008)		Sec4p (Calero and Collins, 2002)
	Rtn4a/b (Voeltz et al., 2006)		Rtn1p (Voeltz et al., 2006)
	—		Rtn2p (Voeltz et al., 2006)
REEP6	Clathrin (Veleri et al., 2017)	—	—
	SNARE/Syntaxin3 (Veleri et al., 2017)		

#### 3.1.2 Yop1p structure/topology

**FIGURE 3:**
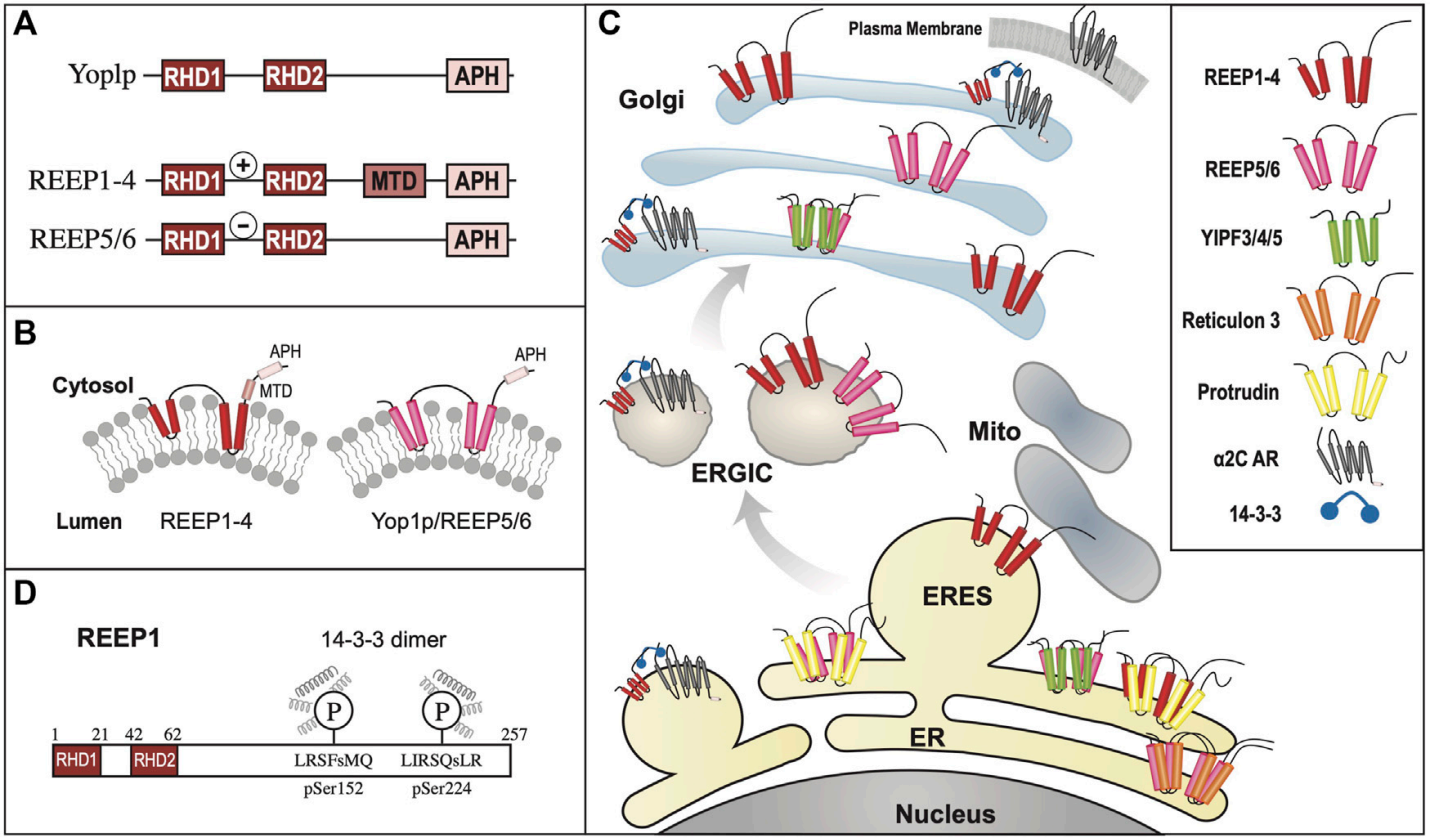
REEP/Yop1p Family of Proteins. **(A).** Domain structure of Yop1p and REEPs based on biochemical analyses (Voeltz et al., 2006; Park et al., 2010). Note that REEP1-4 and REEP5-6/Yop1p have similar structural motif but differ in their carboxy termini (Schlaitz et al., 2013). REEP/Yop1p family members have conserved Rtn homology domains (**RHD**) and a carboxy terminal amphipathic helical domain (**APH**). Additionally, REEP1-4, but not REEP5-6/Yop1p, possess a microtubule-binding domain (**MTD**) between **RHD2** and **APH** (Brady et al., 2015). Additionally, REEP1-4 contain a positively charged region between **RHD1** and **RHD2** that also interacts with microtubules, whereas REEP5/6 possess a negatively charged region that does not interact with microtubules. **(B).** REEP/Yop1p family members have two conserved **RHD** domains that insert into the membrane as hairpin structures and a carboxy terminal **APH** that is either is cytoplasmic or aligned with the membrane (Park et al., 2010). **(C).** Intracellular localization of various REEP family members is shown within the ER, Golgi, and mitochondrial compartments, The role of REEP1 as a membrane-shaping adapter protein (MSAP) is shown with REEP1 binding to a model cargo protein (α2C adrenergic receptor) via an adapter protein (14-3-3 dimer) (Björk et al., 2013). **(D).** REEP1-4 possess conserved multiple potential Ser or Thr phosphorylation sites, that can bind 14-3-3 protein dimers, potential accessory proteins important for cargo trafficking (Tinti et al., 2012). Modeled proteins are not shown to scale relative to their amino acid sequence. ER = Endoplasmic Reticulum, ERES = ER Exit Site, ERGIC = ER/Golgi Intermediate Compartment.

Contemporaneously, two new members of the mammalian reticulon (Rtn) family of proteins were identified, while searching for proteins that shaped the tubular ER (Voeltz et al., 2006). Rtns have a conserved central transmembrane region with two hydrophobic domains (discussed further below). Though Yop1p and Rtn proteins share a low sequence homology, they all possess these two conserved central hydrophobic regions, subsequently termed the “Reticulon Homology Domain” or RHD (Figure 3A). However, utilizing cysteine-substitution mutation analysis, it was suggested that the two “transmembrane” domains described in Rtn and Yop1p RHDs were in fact structured as hairpins, thus making the amino and carboxy termini both cytosolic (Voeltz et al., 2006). Lastly, it was shown that the two short hairpin structures of Rtns insert into the cytoplasmic face of ER membranes to force high curvature, creating ER tubules. Similarly, it was shown that a mammalian homolog of Yop1p, REEP5 (discussed further below) also had a hairpin structured RHD, with cytosolic amino and carboxy termini (Voeltz et al., 2006). In addition, REEP5 was localized to ER membranes at steady-state, consistent with its possible role in ER tubule formation, as seen for Yop1p.

Unlike the Yipf family, whose members appear to contain five potential transmembrane domains, it appeared that Yop1p had only two such transmembrane (TM) domains and initial research suggested that the Yop1p (and its homolog REEP5) formed two hairpins from these domains (Voeltz et al., 2006). However, further analysis by others has suggested that Yop1p actually had five alpha-helical hydrophobic domains (similar to that described originally for Yip1p and Yipf family members), in which the first four of these regions (two RHD domains) formed hairpin structures (Figure 3B). This alternative structure arose from an NMR study of Yop1p (Brady et al., 2015). The proposed model suggested that Yop1p had four alpha helical regions that span the membrane, whereas there was a fifth hydrophobic stretch termed the “amphipathic helical domain” in the extreme carboxy terminus, that did not traverse the membrane. Instead, it laid parallel to and interacted with the negatively charged membrane. Additionally, the APH was necessary for ER tubule formation (Brady et al., 2015). Interestingly, this topological model is similar to the AlphaFold model proposed for the carboxy termini of the mammalian Yipf family), where the APH lies parallel to or buried in the Golgi membrane (Figure 2B) (Calero et al., 2002; Kranjc et al., 2017). Despite the differences between these models, they both depict the amino and carboxy termini of Yop1p and REEP as being cytoplasmic and not intralumenal, as opposed to the early structures proposed for Yipf family members.

So, what is the potential importance of the RHD? In an elegant series of experiments, it was demonstrated that the two hairpin domains within the RHD were required for membrane partitioning and membrane shaping of the ER, thus demonstrating a crucial functional role for this conserved RHD hairpin structure of Rtns, and presumably REEP5/Yop1p (Zurek et al., 2011). Subsequently, it was confirmed that Yop1p could also induce high curvature ER tubules and that this property of Yop1p required only the central portion of the protein containing the two hairpin regions or RHD (Hu et al., 2008). In addition, it was shown that Yop1p formed homo-oligomers in ER membranes, as well as form hetero-oligomers with Rtn1p. Similarly, REEP5 and Rtn4 formed homo- and hetero-oligomers in higher eukaryotes (Shibata et al., 2008). Though the complete family of mammalian Yop1p homologs (the REEP family) had yet to be identified, it appeared that Yop1p had several roles in cell biology including membrane trafficking by binding to Yip1p, SNAREs, and Ypt proteins, as well as inducing ER tubule formation. This last discovery led to the description of these proteins as “ER morphogens,” possibly separate from their role in intracellular transport (Voeltz et al., 2006).

### 3.2 Mammalian REEP family

Previously, the first two members of the mammalian “Yop1p” family had been identified unknowingly, “Deleted in Polyposis 1” or DP1/TB2 and “Deleted in Polyposis 1-like 1” DP1L1/TB1 (Joslyn et al., 1991; Kinzler et al., 1991; Nishisho et al., 1991; Sato et al., 2005), however their connection to Yip1p or Yop1p did not occur until later. These two mammalian orthologs of Yop1p were initially identified while searching for genes implicated in colon cancer pathogenesis. At the time of their initial discovery, yeast Yip proteins had not been identified, so the function of these proteins remained unknown.

However, many laboratories were investigating trafficking of GPCRs from ER to plasma membrane and it was known that many GPCRs (e.g., olfactory receptors, taste receptors) did not express well in mammalian heterologous expression systems. Thus a search for proteins that could enhance heterologous cell surface expression of olfactory receptors (OR) led to the discovery of a new family of six proteins termed “receptor expression-enhancing proteins” or REEP1-6 (Saito et al., 2004). REEP1 was demonstrated to selectively enhance the cell surface expression of some but not all difficult to express ORs, suggesting a specific function for REEP1 dependent upon the “cargo” (e.g., ORs or other GPCRs). Around the same time, a database search of Yop1p-like sequences in mammalian EST and other databases led to the discovery of a new subset of mammalian Yop1p proteins that were alternatively named Yip2a-f (Table 3) (Yang et al., 1998; Pfeffer and Aivazian, 2004). Due to the extensive literature published using the REEP nomenclature, it has superseded the more consistent Yip2 nomenclature, and will be utilized here.

The REEP family was further subdivided, based upon sequence homology, into REEP1-4 and REEP5-6 families (Supplemental Figure S4). The originally cloned DP1/TB2 and DP1L1/TB1 genes belong to the latter subfamily and have been renamed REEP5 and REEP6 respectively; they have the most homology with Yop1p. After cloning REEP1, it was found to be homologous to Yop1p and also a barley stress-induced protein HVA22 (Brands and Ho, 2002). Prior studies of HVA22 revealed that it interacted with a protein termed barley Sey1, a homolog of another plant protein Root Hair Defective 3 (RHD3), an ortholog to the mammalian atlastin family of ER membrane fusion proteins (Park et al., 2010). Similar to Yop1p, the atlastin family of proteins also appear to have a hairpin structure used to insert and shape ER membranes, as well as possessing a conserved APH in the carboxy terminus (Park and Blackstone, 2010). It was shown that the atlastin APH bound to membranes as a membrane-parallel alpha helix, inducing bilayer thinning and increasing acyl chain disorder, leading to membrane destabilization and fusion (Faust et al., 2015). Though their role in ER membrane fusion was not completely characterized, it appeared that REEP family members may be classified as ER morphogens based on research with Yop1p (Voeltz et al., 2006), and that they may have a role in intracellular trafficking and processing of cargo proteins.

#### 3.2.1 REEP cell biology

Yop1p, REEP1, and REEP5 were initially localized to ER membranes by immunohistochemistry, consistent with a possible role for these proteins as ER morphogens (Calero et al., 2001; Saito et al., 2004; Voeltz et al., 2006). Further investigations utilizing membrane fractionation revealed expression of REEP1, REEP2, and REEP6 in ER membranes, more consistent with prior results with Yop1p (Figure 3C) (Björk et al., 2013). Lastly, it was demonstrated by *in vitro* assay, that REEP1-4 are directly involved with ER membrane shaping and tubule formation, similar to REEP5 and Yop1p (Park et al., 2010). Similar ER localization was seen for the multiple *Drosophila* REEP orthologs (Yalcin et al., 2017).

However, other investigators detected REEP1 expression in mitochondria as well (Zuchner et al., 2006). More recent work utilizing a novel split-*R*Luc8 assay demonstrated that REEP1 was present at ER-mitochondria interfaces, demonstrating that different subdomains of REEP1 were required for ER (REEP1^1−115^ = ‘RHD’) and mitochondrial (REEP1^116−157^ = ‘APH’) localization respectively, and that mitochondrial localization was not due to alignment with microtubules (Lim et al., 2015). Furthermore, it was demonstrated that DNA damage-induced changes in ER structure were dependent upon transcriptional activation of REEP1 and REEP2, promoting formation of ER-mitochondrial contacts, facilitating Ca^++^ movement from ER to mitochondria, promoting apoptosis (Zheng et al., 2018). Together, these data suggest a potential role for REEP1 and possibly other REEPs in regulating ER-mitochondrial morphology, signaling and apoptosis. What other role may REEPs play in cell biology? It has been demonstrated that REEP1 can alter lipid droplet size within cells, possibly a function important for ER membrane fusion and tubulization (Falk et al., 2014). Additionally, REEP1 was demonstrated to interact with a protein previously identified as a causative gene for a form of lipodystrophy, seipin (Renvoise et al., 2016). Seipin is a conserved integral membrane ER protein which is believed to act as an interface between the ER and lipid droplets.

Further research in other organisms has suggested that REEPs may suppress autophagy. For example, it has been demonstrated in the plant *Arabidopsis*, that deletion of REEP orthologs (AtHVA22), enhanced autophagy, and a similar effect was seen in Yop1p-deleted yeast (Chen et al., 2009). It was postulated that under stressful conditions, HVA22 was required to inhibit activation of programed cell death. In what may be a similar effect, downregulation of *Drosophila* REEP1 enhanced toxicity from Tau protein by increasing formation of insoluble aggregates. However, this effect could be rescued by overexpression either *Drosophila* or human REEP1 (Appocher et al., 2014). Recently, it has been shown in *Drosophila*, that REEP1 is upregulated under stressful conditions and that the absence of REEP1 led to selective activation of the Ire1 and Atf6 branches of the Unfolded Protein Response (UPR) leading to modification of ER morphology (Napoli et al., 2019).

So, unlike the Yipf family, whose members were identified by sequence homology from genetic databases, REEP family members were identified based upon their function as enhancers of heterologous OR expression, and not strictly by their role in intracellular transport. Additionally, REEP1 and atlastin-1 were identified separately as genes implicated in the development of the neurodegenerative disorder hereditary spastic paraplegia (HSP) (Blackstone et al., 2011). Over fifty percent of North American HSP cases are due to mutations in M1-spastin, atlastin-1, or REEP1. Recent work has shown that M1-spastin, atlastin-1, and REEP1 interact within the ER and appear to be important determinants of curved ER tubule formation and elongation (Hu et al., 2009; Park et al., 2010). All three of these proteins have similar membrane topologies; specifically, they all possess partial membrane spanning hairpin(s) and APH domains.

The hydrophobic hairpin domains of REEPs are necessary for membrane interactions with atlastin-1, M1-spastin, and reticulons (Park et al., 2010). Missense mutations that alter specific amino acids in the hydrophobic hairpin domains have been identified, however the effect of these REEP1 mutations on cargo transport and ER interactions have not been fully elucidated. Similarly, REEP2 has been shown to be a causative agent of HSP and REEP1 has been shown to be a cause of hereditary motor neuropathy (HMN) as well (Beetz et al., 2012; Esteves et al., 2014). Given the neuronal-specific expression of REEP1/2, it is not surprising that loss of REEP function would lead to a neuron-specific disorder (Hurt et al., 2014). Besides HSP, REEP family members have been linked to other genetic and developmental disorders. For instance, REEP3 has been linked to hereditary congenital facial paresis and autism, deletion of REEP4 in *Xenopus* embryo led to neuromuscular paralysis, deletion of REEP5/6 has been seen in familial adenomatous polyposis, and deletion or mutations in REEP6 have been linked to retinal degeneration and retinitis pigmentosa respectively (Groden et al., 1991; Castermans et al., 2007; Argasinska et al., 2009; Tomas-Roca et al., 2011; Arno et al., 2016; Veleri et al., 2017). Thus, initial characterization of REEP family members focused on their function as receptor chaperones or escort proteins, as well as their role in neurodegeneration, and less on their possible roles in intracellular membrane transport.

#### 3.2.2 REEP interacting proteins

Both REEP5 and REEP6 were identified as Rtn4a/b interacting proteins based upon immunoprecipitation and mass spectrometric analysis (Voeltz et al., 2006). Other REEP5 interacting proteins were subsequently identified by others, including Rtn3c and another hairpin protein implicated in HSP, protrudin (Shibata et al., 2008; Chang et al., 2013; Hashimoto et al., 2014). Protrudin was also shown to interact with all three isoforms of atlastin as well as REEP1, and these interactions were dependent upon the membrane spanning region of protrudin, a homologous hairpin region described previously as being necessary for interaction of Rtns, REEPs, Yop1p, and atlastins with each other. Separately, a profile of Rab GTPase trafficking networks (“The Membrome”), suggested that REEP2 and REEP4 both could interact with Rab1b and Rab3a, though this has not been confirmed experimentally (Gurkan et al., 2005). Similar to Yop1p, REEP1 could form high order oligomers, suggesting self-interaction, however, interactions between different REEPs have not been published (Park et al., 2010). Recently, REEP6 has been shown to interact with a t-SNARE protein Syntaxin3, as well as being expressed in a subset of clathrin-coated vesicles in retinal photoreceptor cells (Veleri et al., 2017).

#### 3.2.3 REEP structure/topology

The original membrane topology of REEPs and Yop1p was not settled (Figure 3B). Initial research with REEP5 suggested a two hairpin model with cytoplasmic facing amino and carboy termini (Voeltz et al., 2006), however, a more recent study with Yop1p suggested five hydrophobic/transmembrane domain, with the most carboxy terminal transmembrane domain laying on, and not inserted into, the membrane, as an APH. The possible evolutionary importance of the APH domain was further demonstrated when a retinal specific splice variant of REEP6 was shown to include a carboxy terminal 27 amino acid APH domain (encoded by Exon 5), that is spliced out in other cell types where REEP6 is expressed (Liang et al., 2021). The first two “transmembrane” domains actually form dual hairpins that insert into the membrane (Brady et al., 2015). Protease analysis further supported a two hairpin model for REEP1, revealing that both the amino and carboxy termini are cytoplasmic, consistent with earlier work with REEP5 (Park et al., 2010). Further evidence for this topological model can be seen by examining the AlphaFold database, which predicts a dual hairpin structure with a carboxy terminal alpha-helical domain (APH) for all six REEP family members (Supplemental Figure S3) (Jumper et al., 2021; Varadi et al., 2022). Alternatively, a single transmembrane model of REEP1 in the plasma membrane with extracellular amino terminus and intracellular carboxy termini, has been proposed. However the localization of this model to the plasma membrane and the single transmembrane model itself are inconsistent with prior research (Ilegems et al., 2010).

Subsequently, it was shown that overexpressed REEP1 altered ER morphology, revealing a distribution of REEP1 in ER tubules closely aligned with thickened microtubules; this discovery was also demonstrated with REEP2 (Park et al., 2010). However, similar findings were not seen with either REEP5 or REEP6. *In vitro* microtubule-binding assays demonstrated that REEP1 could immunoprecipitate tubulin and that the region of interaction was the cytoplasmic carboxy terminus of REEP1, revealing another protein interacting domain within REEP1. The exact amino acid sequence of the microtubule-binding domain (**MBD**) has not been identified (Figure 3A). Given the homology between REEP1-4, it seems likely that the **MBD** may be conserved in this subfamily of REEP proteins.

A different microtubule binding region within REEP3/REEP4 was identified when it was discovered that REEP3/REEP4 were important for clearance of ER membranes from metaphase chromatin, to ensure correct progression through mitosis (Schlaitz et al., 2013). The region of microtubule interaction was located between the two hairpin domains, specifically a positively charged cytoplasmic region that is conserved in REEP1-4, unique from the **MBD** domain within the carboxy terminus, described above. Interestingly, the corresponding region within REEP5/6 is negatively charged and did not interact with microtubules (Figure 3A).

While looking for proteins that contained binding sites for the family of 14-3-3 proteins, another major protein interaction domain was discovered in REEPs (Johnson et al., 2011). 14-3-3 proteins are intracellular adapter proteins that interact specifically with phosphoproteins, usually by interacting as a 14-3-3 dimer with two tandem phosphorylated sites within the target protein (Johnson et al., 2010). These phosphorylated 14-3-3 binding sites on target proteins are phosphorylated by protein kinase A, protein kinase C, protein kinase G, Ca^2++^/calmodulin-dependent protein kinase (CaMK) and potentially other kinases as well, demonstrating a potentially complex level of regulation by multiple kinase inputs.

While examining the proteome for 14-3-3 interacting proteins, Johnson et al., identified and biochemically verified that REEP4 contained two phosphorylatable 14-3-3 binding sites within its carboxy terminus (Figure 3D) (Johnson et al., 2011). Subsequently they demonstrated that REEP1-4, but not REEP5-6/Yop1p possess such 14-3-3 binding sites (Tinti et al., 2012). The binding of 14-3-3 proteins requires two sites of phosphorylation, and it was shown that REEP1-4 all share a common first phosphorylation site (e.g., pSer152 in REEP1), termed the “lynchpin” phosphorylation site, however they also possess a unique second phosphorylation site (e.g., pSer192 in REEP1), which appear to be phosphorylated by different kinases, leading to differential affinities for 14-3-3 dimers amongst REEP1-4 proteins. Possible Ser, Thr, and/or Tyr phosphorylation sites have been identified in all REEPs. The role of phosphorylation in REEP family function has not been studied, however, removal of all potential phosphorylation sites from REEP1 led to an inability of the expressed protein to form higher order oligomers with itself (unpublished data).

#### 3.2.4 REEP cargo trafficking

The REEP family was identified by its ability to enhance heterologous cell surface expression of GPCRs, that were difficult to express. Their effects on native expression of ORs or other GPCRs had not been investigated. Initially, REEP1 enhancement of heterologously expressed ORs was examined, and it was determined that this effect was restricted to a subset ORs, though it was not determined what identified this subset (Saito et al., 2004). However, similar to ORs, it was shown that REEP1 could enhance the heterologous expression of bitter taste receptors (TAS2R) (Behrens et al., 2006). Other similar receptors, sweet taste receptors (T1R2/T1R3), where investigated with REEP2, and a novel mechanism of action was described, specifically that REEP2 enhanced sweet receptor function by their recruitment to lipid rafts (Ilegems et al., 2010). Further research with respect to REEPs and lipid rafts has not been described.

The selective interaction of GPCRs with REEPs was investigated further by comparing the effects of co-expressed REEP on two model cargo proteins, α2_A_ and α2_C_ adrenergic receptors (ARs) (Björk et al., 2013). These two cargos were chosen since they were highly homologous proteins yet had differing levels of heterologous expression in various cell lines. Specifically, heterologous expression of α2_A_ARs demonstrated higher levels of plasma membrane expression, compared to the more difficult to express α2_C_ARs (Daunt et al., 1997). Similar to ORs, co-expression of either REEP1, REEP2, or REEP6 led to enhanced plasma membrane expression of α2_C_ARs, but did not affect plasma membrane expression of α2_A_ARs, suggesting specificity of REEP family members for cargo protein. By utilizing a FACS-based single cell assay (Hurt et al., 2013), it was possible to quantify both plasma membrane and intracellular levels of each cargo protein concurrently, and it was shown that all three REEPs studied enhanced trafficking of α2_C_ARs to the plasma membrane by enhancing cargo capacity of the ER (Figure 3C). Specifically, immunoprecipitation experiments demonstrated that all three REEPs interacted with α2_C_ARs, but not α2_A_ARs, by interacting specifically with a minimally/non-glycosylated form of α2_C_ARs, demonstrating that REEPs could selectively interact and alter cargo protein trafficking through the cell. Identification of the minimally/non-glycosylated form of α2_C_ARs was reminiscent of work with CPY and Yop1p, where it was shown that overexpression of Yop1p in yeast led to an accumulation of minimally/non-glycosylated form of CPY in the ER (Calero et al., 2001). Lastly, it was shown that this interaction required the carboxy terminus of REEP1 (including the MTB and 14-3-3 binding sites), suggesting a role for this region in cargo protein trafficking. Thus, REEPs appeared to have additional intracellular functions besides being merely ER morphogens; co-expressed REEPs enhanced cargo capacity of a cell, affected ER-Golgi glycosidic processing, and interacted with specific cargo proteins.

The specific site or interacting domain of REEPs responsible for cargo binding has not been identified, except to demonstrate that it lies within the cytoplasmic carboxy termini, where variable phosphorylation, MTB, APH, and 14-3-3 binding sites are known to exist. Thus, REEP family members could either directly interact with cargo or utilize adapter proteins. A strong contender for a REEP adapter would be the 14-3-3 family of proteins. First, REEP1-4 all have a conserved 14-3-3 binding site in the COOH terminus (RSXpS or RXXXpS, where pS represents phosphoserine), interestingly, REEP5-6 do not have this conserved motif (Morrison, 2009). Second, it was demonstrated that phosphorylation of REEP4 at this site increased 14-3-3 binding, however mutation of this serine did not completely abolish 14-3-3 binding. However, complex interplay of various kinases on the multiple potential phosphorylation sites may account for the lack of complete effect of the mutation. The identification of other adapter proteins may require further study and analysis of other protein-protein interacting domains within the Yip family.

### 3.3 Yop1p/REEP family summary

Unlike the Yipf family, where most of the research followed the identification of mammalian orthologs to yeast Yip1p proteins and paralleled their characterization within mammalian cells, REEP family members were initially identified and characterized based upon their function as enhancers of GPCR expression, as genetic causes of HSP, and as 14-3-3 interacting proteins. However, these different avenues of identification of the REEP family led to more information about their structure, function, interacting proteins, and potential regulation by phosphorylation. It is apparent that Yop1p and REEP family members are ER resident proteins, and they all possess two RHD domains (analogous to the “Yip Domain” of Yipf family members), structured as dual hairpins that insert within ER membrane to force high curvature and ER tubulization. Yop1p has been shown to interact with other proteins involved with intracellular membrane transport, including other Yip family members, COPII proteins, and Ypts, though more complete analysis of similar REEP interactions with transport machinery proteins remain to be done. However, it has been shown that REEP4 can interact with specific Rabs or other Rab effectors (RAB3GAP-1/2), consistent with a role in regulating membrane trafficking by affecting Rab-GTPase cycling (Tinti et al., 2012). But again, no specific roles for REEPs/Yop1p in Rab cycling (e.g., GEF, GAP, GDI, GDF) have been identified (Figure 1).

Unlike Yipf family members, more is known about different structural domains with REEP family members, but several areas of uncertainty remain. Strong evidence suggests the presence of two hairpin domains (the RHD); however, sequence analysis suggests five alpha-helical domains in these proteins (the first four encoding two RHD domains) and thus the position of the fifth carboxy terminal domain remains to be determined. Strong experimental evidence in Yop1p and REEP5 suggests that this domain behaves as an APH that aligns parallel to, and does not insert into, the membrane. This domain that may also be necessary for ER membrane insertion and induction of high membrane curvature. Beyond the RHD, microtubule binding to an MTB domain has been identified within the cytoplasmic carboxy terminus of REEP1, and based upon homology, to REEP1-4, but the exact MTB sequence remains to be determined. Lastly, phosphorylation sites for 14-3-3 dimer binding has been identified in REEP1-4 but not REEP5-6/Yop1p, but which kinases phosphorylate which REEPs, and what effect phosphorylation has on any of the described REEP functions have not been completely elucidated. Thus, it appears that Yop1p/REEP1-6 have similar topology with two RHD hairpin domains and a carboxy APH lying on the membrane, separated by a charged region (++ = REEP1-4, -- = REEP5-6), MTB and APH domains, as well as potential phosphorylation and 14-3-3 protein binding sites (Figure 3A/D).

When REEP1 was identified as a causative gene for HSP, its interactions with other genes involved with HSP was characterized, including the atlastin family, M1-spastin, and protrudin (Park and Blackstone, 2010; Hashimoto et al., 2014). It is now known that REEP1 can interact with these proteins, and that this interaction depends upon the presence of the RHD. Though a review of the genetics of HSP and role of REEP1 and the other mentioned proteins is beyond the scope of this review, it is apparent that loss of REEP1 function can have major effects of ER structure and neuronal function, eventually leading to neurodegeneration of spinal cord motor neurons and development of HSP (Blackstone, 2018a). To date, over twenty mutations in REEP1 have been linked to HSP (Zuchner et al., 2006; Schlang et al., 2008; Liu et al., 2009). These mutations include missense and nonsense mutations, deletions, and frameshifts. In most cases, the frameshift mutations lead to a premature truncation of the protein, and thus deletion of the carboxy terminal region, possibly affecting MTB, APH, 14-3-3 and potential phosphorylation sites or other binding domains. The extensiveness of REEPs/Yop1p research, from cell biology, receptor trafficking, protein-protein interacting and phosphorylation domains, and disease genetics, coupled with the multiple experimental organisms studied, has led to a more complete understanding of this Yip subfamily.

## 4 Yeast/mammalian Yif family

### 4.1 Yeast Yif1p

Another member of the larger yeast Yip family, the Yip1p-interacting factor or Yif1p, was identified by Y2H screening while searching for other yeast proteins that bound Ypts (Table 5) (Matern et al., 2000). Similar to Yip1p, Yif1p appeared to possess five transmembrane domains, with an elongated amino terminus and a truncated carboxy terminus. It was further demonstrated that Yif1p bound Yip1p, Ypt1 and Ypt31 (similar to Yip1p) (Table 6) and that Ypt binding was dependent upon the amino terminus of Yif1p, whereas Yip1p binding did not require this region. Lastly, the elongated amino terminus was cytosolic. In fact, a majority of the protein’s amino terminus could be deleted (up to the second transmembrane domain) without loss of Yip1p interaction. Yif1p and Yip1p could both reciprocally immunoprecipitate each other. Other interacting proteins discovered in yeast included Sec4p, a Rab GTPase involved with the final exocytic secretory pathway from Golgi to plasma membrane. Separately, it was demonstrated that Yif1p could be found within COPII vesicles (Otte et al., 2001). As mentioned earlier for Yip1p, Yif1p was required to produce ER-derived fusion competent vesicles for fusion with Golgi membranes (Barrowman et al., 2003). Additionally, Yos1p was also identified as an interacting protein for Yif1p (Heidtman et al., 2005).

**TABLE 5:** Mammalian Yif1 and PRAF families.

HGNC	Alternative nomenclature (Shaik et al., 2019)	Alternative names	Yeast homolog	TM/HP domains	Localization	Cargo
Yif1A (Yoshida et al., 2008)	YIPFβ1A	HuYif1 (Jin et al., 2005)	Yif1p (Matern et al., 2000)	5	Golgi (Shakoori et al., 2003)	
		FinGER7 (Shakoori et al., 2003)			ER-Golgi Tx (Kuijpers et al., 2013)	
		—			ERGIC (Yoshida et al., 2008; Kuijpers et al., 2013)	
Yif1B (Yoshida et al., 2008)	YIPFβ1B	FinGER8 (Shakoori et al., 2003)	—	5	Golgi (Shakoori et al., 2003)	5HT_1A_R (Carrel et al., 2008)
					ER-Golgi Tx (Carrel et al., 2008)	
					ERGIC (Carrel et al., 2011; Alterio et al., 2015)	
PRAF1 (Fo et al., 2006)	—	Yip3 (Sivars et al., 2003)	—	4	—	
		PRA1 (Pfeffer and Aivazian, 2004)	Yip3p (Calero and Collins, 2002)		Golgi (Abdul-Ghani et al., 2001)	
		RABAC1 (Abdul-Ghani et al., 2001)	PRA1p (Calero and Collins, 2002)		Endosome (Sivars et al., 2003; Geng et al., 2005)	
		Prenylin (Liang et al., 2004)	—		—	
PRAF2 (Fo et al., 2006)	—	Yip6a (Ruggiero et al., 2008)	—	4	ER (Abdul-Ghani et al., 2001; Fo et al., 2006)	CCR5 (Schweneker et al., 2005)
		PRA3 (Pfeffer and Aivazian, 2004)				GABA_B1_R (Doly et al., 2016)
		JM4 (Schweneker et al., 2005)				
PRAF3 (Fo et al., 2006)	—	Yip6b (Ruggiero et al., 2008)	—	4	ER (Abdul-Ghani et al., 2001; Ruggiero et al., 2008)	EAAC1 (Lin et al., 2001a; Ruggiero et al., 2008)
		PRA2 (Pfeffer and Aivazian, 2004)				EAAT1-4 (Butchbach et al., 2002; Ruggiero et al., 2008)
		JWA (Wu et al., 2011)				β_2_AR, α_2B_AR, D_2_R (Ruggiero et al., 2008)
		GTRAP3-18 (Butchbach et al., 2002)				DOR (Wu et al., 2011)
		Arl6-IP5 (Ingley et al., 1999)				CCR7, 5HT_2_R, CCR2 (Doly and Marullo, 2015)
		Addicsin (Inoue et al., 2005)				

**TABLE 6:** Yif and PRA family interacting proteins.

Mammalian	Mammalian interacting proteins	Yeast	Yeast interacting proteins
Yif1A	—	Yif1p	Ypt1/31p (Matern et al., 2000)
	—		Yip1p (Matern et al., 2000)
	Yipf5 (Shakoori et al., 2003; Jin et al., 2005)		Yos1p (Heidtman et al., 2005)
	VAMB (Kuijpers et al., 2013)		Sec4p/VPS21 (Matern et al., 2000)
	—		COPII Vesicles (Otte et al., 2001)
Yif1B	Yipf5 (Al Awabdh et al., 2012)	—	—
	Rab6 (Al Awabdh et al., 2012)		
	Kif5B (Al Awabdh et al., 2012) dynein (Al Awabdh et al., 2012)		
	α2/β2 tubulin (Al Awabdh et al., 2012)		
PRAF1	Rab4B/5A/5C (Bucci et al., 1999)	Yip3p	—
	Rab1/3A (Martincic et al., 1997; Hutt et al., 2000)		—
	Rab5/7/9 (Sivars et al., 2003)		Ypt1p (Geng et al., 2005)
	VAMP2 (Martincic et al., 1997)		Yip1p (Calero and Collins, 2002)
	ζ1-COP (Abu Irqeba and Ogilvie, 2019)		Rtn1p (Geng et al., 2005)
	γ-COP (Lee et al., 2011)		COP II Vesicles (Otte et al., 2001)
	Piccolo (Fenster et al., 2000)		—
	BCL2A1 (Kim et al., 2019)		—
PRAF2	PRAF3 (Schweneker et al., 2005)	—	—
PRAF3	PRAF2 (Schweneker et al., 2005)	—	—
	Arl6IP1 (Yamamoto et al., 2014)		
	Rtn2B (Liu et al., 2008)		

#### 4.1.1 Yeast Yif1p cell biology

Similar to Yip1p, Yif1p was originally localized to Golgi membranes by both indirect immunofluorescence and sucrose gradient fractionation (Matern et al., 2000). Loss of Yif1p function was not lethal and led to a block in ER to Golgi transport, with accumulation of ER membranes as had been seen previously with other Yipf and REEP family members. More specifically, loss of Yif1p affected the processing of a model protein CPY, leading to the accumulation of an ER core-glycosylated form of CPY, as was seen with Yop1p deletion. Lastly, high exogenous expression of Yif1p could rescue Yip1p mutant forms (Calero et al., 2001).

### 4.2 Mammalian Yif family

#### 4.2.1 Mammalian Yif cell biology

**FIGURE 4:**
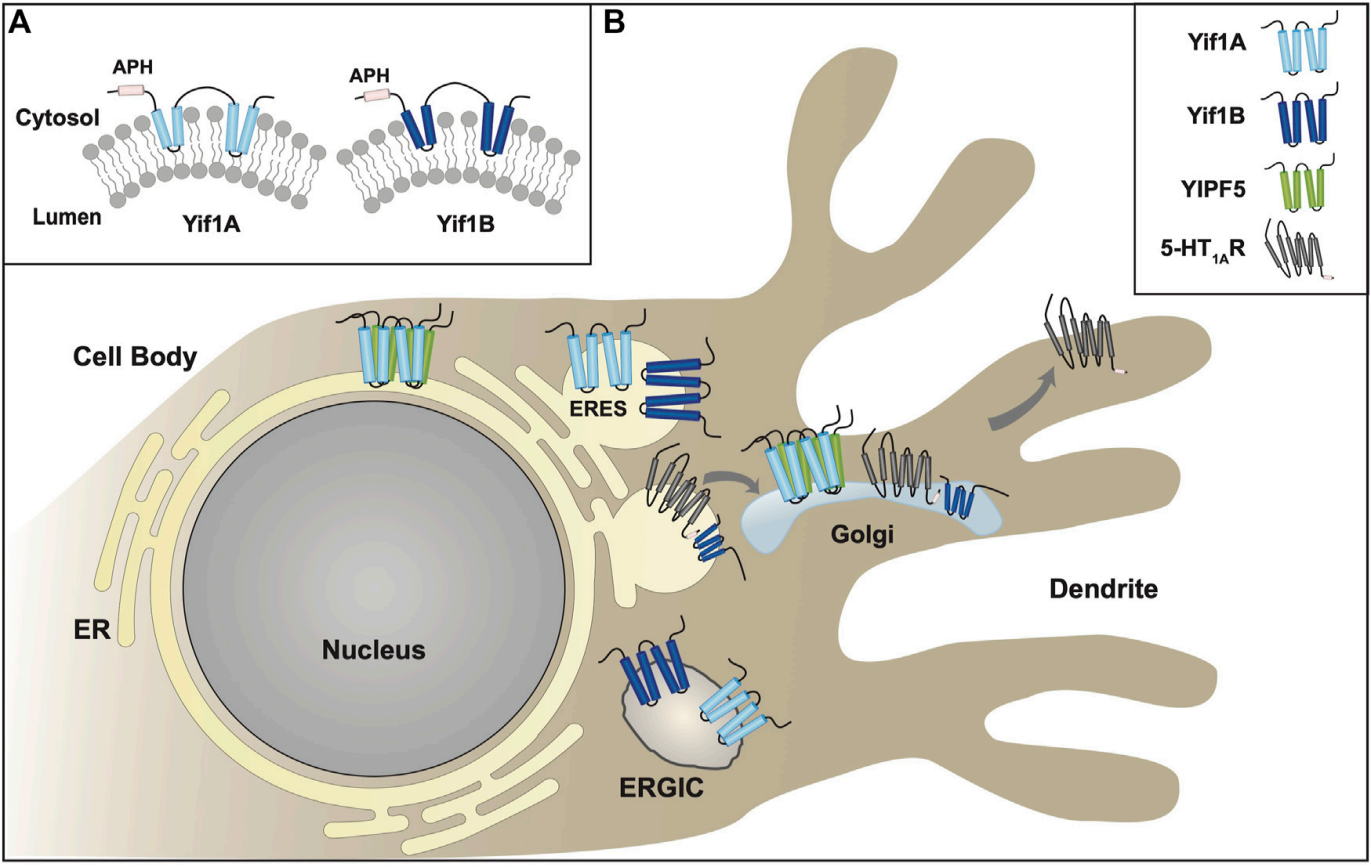
Yif1/Yif1p Family of Proteins. **(A).** Transmembrane topology model of Yif1 and Yif1p based on biochemical analyses (Matern et al., 2000; Al Awabdh et al., 2012). Unlike Yipf and REEP family members, the exact Yif1 membrane topology has not been delineated but is shown as hairpin domains due to Yipf family homology. Unlike Yipf and REEP families, an APH domain has been found within the amino terminus. **(B).** Intracellular localization of various Yif family members is shown within the ER, Golgi, and ERGIC compartments, as well as transport machinery required for Yif1 cargo trafficking within neurons. The role of Yif1B as a regulator of cargo trafficking (and possible MSAP) is shown, interacting with a well-described cargo protein (5-HT_1C_ receptor) (Carrel et al., 2011). Note the absence of an adapter protein. Modeled proteins are not shown to scale relative to their amino acid sequence. ER = Endoplasmic Reticulum, ERES = ER Exit Site, ERGIC = ER/Golgi Intermediate Compartment.

As mentioned above, nine FinGER proteins had been originally cloned as mammalian orthologs to Yip1p, Yip4p, and Yip5p. Two of these proteins, FinGER7-8, shared the most homology with Yif1p and were renamed Yif1A and Yif1B respectively (Table 5, Supplemental Figure S5) (Shakoori et al., 2003). Similar to Yif1p, Yif1A and Yif1B appeared to possess five transmembrane domains, with an elongated amino terminus and a truncated carboxy terminus (Figure 4A). However, the first alpha helical region was found in the amino terminus, followed by four more transmembrane domains, as compared to REEP/Yop1p which had two hairpin domains/four transmembrane domains with a carboxy terminal alpha helical domain. Previously, the membrane topology of seven Yipf family members was determined utilizing a fluorescence protease protection assay (Kranjc et al., 2017). Applying a similar topology model to the related Yif1A/B proteins, would suggest that the amino terminus, not the carboxy terminus, was intralumenal or possibly buried in the Golgi membrane as an APH domain (Figure 4A). This model is predicted by AlphaFold (Supplemental Figure S3) (Jumper et al., 2021; Varadi et al., 2022).

Initial characterization of Yif1A and Yif1B suggested that they were located to the Golgi, similar to Yif1p (Shakoori et al., 2003). However, other investigations suggested a more dynamic process of localization, demonstrating that they are localized to both ER and Golgi or the ERGIC, potentially reflecting their role in ER-Golgi transport via COPII vesicles insertion (Figure 4B) (Kuijpers et al., 2013). Subsequently it was demonstrated that Yif1A could cycle between ER and Golgi within hippocampal neurons, and it was mainly localized to the ERGIC (Yoshida et al., 2008). Analysis of Yif1B also demonstrated a similar ER, ERGIC, and Golgi localization (Shakoori et al., 2003; Carrel et al., 2008). Interestingly, it was demonstrated that depletion of Yif1B led to accelerated intracellular trafficking of a model protein, VSVG, from ER to ERGIC to Golgi within hippocampal neurons of Yif1B-KO mice (Alterio et al., 2015). Either way, it appeared that similar to Yipf family members, Yif1A and Yif1B cycle between ER and Golgi membranes, including the ERGIC. Knockdown of Yif1A did lead to partial disassembly of the Golgi apparatus, as was seen with Yipf5, and Yif1B deletion lead to ER disorganization in mouse hippocampal neurons and cerebellar Purkinje cells (Yoshida et al., 2008; Diaz et al., 2020).

#### 4.2.2 Mammalian Yif interacting proteins

Few interacting proteins have been identified to date for Yif1A or Yif1B (Table 6). They have been shown to directly interact with other Yipf family members, specifically Yipf5, as seen by immunoprecipitation (Shakoori et al., 2003; Jin et al., 2005). Deletion of the carboxy terminus of Yipf5 disrupted the Golgi localization of Yif1A, suggesting that Yipf5 specified the localization of Yif1A. However, other interacting proteins have been identified that also may impact localization of Yif1A, namely “vesicle-associated membrane protein (VAMP) associated protein B” or VAPB (Kuijpers et al., 2013). VAPB is an ER resident protein, and a single mutant allele of this protein has been linked to amyotrophic lateral sclerosis. It was demonstrated that overexpressed VAPB can bind Yif1A in the ER and thereby prevent its recycling to the ERGIC and Golgi. Further investigations in hippocampal neuron cultures revealed that Yipf5, Yif1A/B, and VAPB were required for normal dendrite morphology and that Yif1A/B were required for intracellular delivery of membrane from soma to dendrite via the early secretory pathway. Additionally, VAPB was required for intracellular membrane of trafficking of Yif1A into dendrites and for maintaining normal dendritic morphology.

#### 4.2.3 Mammalian Yif cargo trafficking

Though some Yip family members have been linked to neurodegenerative disorders (e.g., HSP), much research has been focused on model cell systems such as yeast or cell lines, and not neurons. As described above, interacting cargo proteins associated with various Yip family members have been identified for REEP family members (e.g., ORs, TRs, and α2_C_ARs). However, trafficking of 5-HT_1A_ serotonin receptors (5HT_1A_Rs) has been studied extensively due to its specific localization within the somatodendritic domain of neurons. Its localization to the dendritic membrane is closely related to its inhibiting effect on raphe neuron firing, and thus mechanisms responsible for this specific localization have been investigated. Prior research had shown that a dileucine motif within the short cytosolic carboxy terminus of 5HT_1A_R was necessary for export from the ER to the plasma membrane (Carrel et al., 2006).

Utilizing the carboxy terminus of 5HT_1A_R as bait, a yeast two-hybrid screen identified Yif1B as an interacting protein (Carrel et al., 2011). This interaction was confirmed by using glutathione-S-transferase pull-down experiments with rat brain extracts and transfected cell lines, demonstrating an *in vivo* interaction between these two proteins. Yif1B was further localized to the ERGIC, similar to other studies with Yif1A and Yif1p (Figure 4B). Lastly, inhibition of Yif1B expression in primary hippocampal neuron cultures led to restriction of 5HT_1A_R expression to the proximal portion of dendrites and not to the distal ends of dendrites, as seen normally. Furthermore, knockdown of Yif1B expression did not alter α-tubulin localization, nor did it alter the dendritic localization of other potential cargo proteins such as the somatostatin receptor (sst2AR), purinergic receptor P2X_2_, or the serotonin ion channel (5-HT_3_R). Lastly, expression of a 5HT_1A_R lacking the carboxy terminal region that interacted with Yif1B, led to its localization in the proximal dendritic compartment, similar to that seen with full-length 5HT_1A_Rs when Yif1B expression was reduced. Taken together, this work was the first to reveal a role for a specific Yip family member in proper neuronal trafficking and localization of a cargo protein.

The specificity of the interaction between Yif1B and 5HT_1A_R was further demonstrated when it was shown that Yif1B could bind the carboxy terminus of 5HT_1A_R with high affinity (K_D_ ≅ 37 nM) via a tribasic motif in 5HT_1A_Rs and a triacidic region within the amino terminus of Yif1B (Al Awabdh et al., 2012). As well, Yif1B was shown not to interact with the closely related 5HT_1B_R, similar to the specificity seen for some REEP family members and α2 ARs (Björk et al., 2013). The concept of Yif family members as scaffold or adapter proteins was further supported when it was determined that Yif1B could form a protein complex with Rab6, α2/β2 tubulin, Yipf5, and two molecular motors, Kif5B and dynein (Al Awabdh et al., 2012). Knockdown of either Rab6, Kif5B, or Yipf5 also led to confinement of co-expressed 5HT_1A_Rs to the proximal dendritic compartment, as seen previously with reduced expression of Yif1B. Lastly, it was shown that intracellular vesicles containing the Yif1B scaffolding complex described above were transported along a microtubule network by two opposing molecular motors, Kif5B and dynein.

Together, these results suggest that targeting of 5HT_1A_Rs to distal dendrites involved a novel vesicular scaffolding-dependent transport pathway, the most complete pathway described for targeting of a cargo protein by a Yipf family member (Figure 4B). Furthermore, examination of Yif1B-knockout mice revealed a significant decrease in forebrain serotonergic projection fibers and reduced function of 5HT_1A_R autoreceptors in raphe serotonergic neurons, possibly responsible for increased social anxiety/less social interaction seen in Yif1B-knockout compared to wild-type mice (Martin et al., 2020).

### 4.3 Yif1p/Yif family summary

Similar to the Yipf family, both members of the Yif family appear to be involved with ER to Golgi cycling, and reside in the requisite intracellular compartments, specifically the ER, Golgi, and ERGIC. This similarity is not too surprising given the overall homology between these two families. Similar to Yipf family members, Yif1p, Yif1A, and Yif1B have been shown to interact with other members of the greater Yipf family, as well as Ypts and Rabs. Thus, it appears that the Yif1 family may have a similar role in regulating intracellular trafficking of proteins. Again, no specific role in Rab cycling (e.g., GEF, GAP, GDI, GDF) has been identified (Figure 1).

Unlike prior research on Yipf and REEP family members, a more complete analysis of Yif1B effects in neurons, revealed that it served as a scaffold protein to link intracellular vesicle trafficking of a cargo protein (5HT_1A_Rs) to their location within the distal dendrite tips. More importantly, it was demonstrated that this Yif1B-containing vesicle could interact with opposing molecular motors (Kif5B and dynein) via binding to tubulin proteins, to move them to and from the distal dendrite. This interaction between Yif1B and its cargo, 5HT_1A_R, was of high affinity and specific amino acid motifs involved in this interaction were determined for both Yif1B and 5HT_1A_R. Truncating mutations in Yif1B have been linked to Kaya-Barakat-Masson syndrome (KABAMAS), a severe autosomal recessive neurodevelopmental disorder characterized by profoundly impaired global development with variable motor abnormalities, as well as another cohort of families with neurological deficits that could not be classified to a specific pathology except that the symptoms seen were consistent with a “Golgipathy” (AlMuhaizea et al., 2020; Diaz et al., 2020).

Compared to other members of the Yipf family, there are still areas to be explored. For example, though Yif1p can be found in COPII vesicles, this finding has not been confirmed for either Yif1A or Yif1B. However, given that one of their binding partners (Yipf5) does bind Sec23/24 (both COPII vesicle proteins), and Yif1A/Yif1B are found in the ER and Golgi, where COPII vesicles arise, it would not be surprising to find Yif1A or Yif1B in COPII vesicles as well. Except for the determination that the larger amino terminus of Yif1p is cytosolic, the remaining structure and topology of any yeast or mammalian Yif1 family member remains to be determined. Based on the originally cloning data, it has been suggested that Yif1 family has five transmembrane domains; APH domains have not been investigated within Yif family members. However, it is more likely that Yif1A/B share a similar topology as Yipf family members, given their homology (FinGER1-9), however Yif1A/B appear to have an amino terminal APH as opposed to a carboxy terminal APH (Shakoori et al., 2003; Kranjc et al., 2017).

Similar to REEPs, it has been demonstrated that Yif1B can interact and direct the location of specific cargo (5HT_1A_Rs) via a larger scaffolding complex made up of Rab6, Yipf5, and Yif1B. This description would be consistent with a model of Yip family members as membrane-shaping adapter proteins (MSAPs) (Bauer and Pelkmans, 2006). As mentioned earlier, MSAPs have been defined by their ability to localize to a specific membrane type(s), alter membrane structure, interact with other proteins via specific domains, and show specificity in their interactions and effects on cargo proteins. Yif1B meets all the criteria except it has not been proven that it can alter membrane structure, in a manner analogous to REEPs or Yop1p. Given the overall homology between these different families, the common finding that the hydrophobic membranous regions of Yipf and REEP family members are important for protein-protein interactions within and amongst the different families, and that this hydrophobic region of Yop1p/REEPs inserts in the membrane as a hairpin, it would not be unreasonable to believe that Yif (and probably Yipf) family members share similar hairpin structures that have been shown to alter membrane structure.

## 5 Yeast Yip3p/mammalian PRAF family

Unlike the prior Yip families, the first member of the Yip3p/PRAF family, PRAF1, was identified in higher eukaryotes, while searching for mammalian proteins that interacted with Rab3A (Martincic et al., 1997). It was shown that the newly discovered protein had two hydrophobic domains, and that it could only bind prenylated Rab proteins, hence the name “Prenylated Rab Acceptor 1” or PRA1 (eventually renamed PRA Family or PRAF). As well, it was demonstrated that PRAF1 also interacted with a synaptic vesicle protein, VAMP2/Synaptobrevin (a t-SNARE protein) but had no affinity for VAMP1 or other Ras-like GTPases. Lastly, Rab- and VAMP2-interacting amino acid residues of PRAF1 were localized to the amino terminus and extreme carboxy terminus respectively. Sequence and topology analysis suggested that the amino terminus was cytoplasmic, with a shorter carboxy terminus that may also be cytoplasmic. However, this model was not tested. Based on these findings, it was suggested that PRAF1 may link Rabs and SNARE proteins (e.g., VAMP2) in order to control synaptic vesicle docking and fusion. PRAF1 subcellular localization was examined in a subsequent study where it was shown that PRAF1 co-localized to the Golgi, and not the ER, however a significant amount of PRAF1 was surprisingly cytosolic, and not membrane bound, contrary to its identification as an integral membrane protein. Lastly, a carboxy terminal truncation mutant of PRAF1 did reside in the ER only, suggesting possible shuttling between the two compartments (Hutt et al., 2000).

### 5.1 Yeast Yip3p

In the original paper describing PRAF1, a related yeast protein Yip3p was identified. This protein was subsequently found to be a component of COPII vesicles; however it was not further characterized with respect to its function (Otte et al., 2001). Subsequently, an initial analysis of Yip3p demonstrated that it appeared to interact non-specifically with multiple Ypt proteins, however it did have a specific interaction with Yip1p (Calero and Collins, 2002). Further work in yeast demonstrated that Yip3p was localized to Golgi and endosomal membranes, as well as the cytoplasm, similar to results seen with PRAF1 (Geng et al., 2005). Additionally, Yip3p was shown to interact with Yip1p, Ypt1p, and Rtn1p *in vivo* and overexpression of Yip3p lead to expansion of the ER.

#### 5.1.1 Yip3p/PRAF1 and rab cycling

Given its role as a ‘Prenylated Rab acceptor’, the nature of PRAF1 function in Rab membrane cycling was investigated further. Prior work had suggested that Rab interactions with a known Rab-GDI, GDI1, is necessary for membrane extraction of the Rab-GDP (Figure 1) (Dirac-Svejstrup et al., 1994; Hutt et al., 2000). Thus, it was subsequently shown that PRAF1 and GDI1 did interact directly, and truncation of the carboxy terminus of PRAF1 abolished this interaction. Since it was known that GDI1 binding to Rab3A could extract Rab3A from the membrane, the effect of PRAF1 on Rab3A extraction by GDI1 was examined. It was demonstrated that the presence of excess PRAF1 inhibited Rab3A membrane extraction by GDI1 resulting in membrane retention of the Rab-GDP (Hutt et al., 2000). Thus, GDI1 and PRAF1 appear to interact competitively for Rab3A binding.

If PRAF1 or Yip3p were involved with Rab cycling between membranes, then it would be possible that PRAF1/Yip3p were identified in the cytoplasm as the Rab-GDP/PRAF1 or Ypt-GDP/Yip3p complex. One proposed hypothesis was that PRAF1/Yip3p functioned as a GDI-displacement factors or GDF, allowing for Rab-GDPs to be returned to their cognate membrane (Figure 1). Evidence for a GDF function for PRAF1 was demonstrated biochemically, when it was shown that it could act catalytically to disassociate Rab9, but not Rab1A, from binding to GDI, and also lead to an increase in GDP-GTP exchange on Rab9 (Sivars et al., 2003). Lastly, depletion of PRAF1 within cells led to a redistribution of Rab9 from membranes to the cytosol, but did not alter Rab1A localization, suggesting a specificity for Rab interaction. Surprisingly, similar studies depleting Yip3p in yeast did not lead to any change in membrane localization for any Ypt examined (Geng et al., 2005). These discoveries suggested that some Yip family members may function as GDF and may be important regulators of membrane trafficking machinery. Similar experiments with Yipf2 and Rab5, Rab8, and Rab22a remain to be performed (Qi et al., 2019; Wang et al., 2020).

### 5.2 Mammalian PRAF family

#### 5.2.1 PRAF cell biology and interacting proteins

Eventually, three PRAF isoforms (PRAF1-3) were identified in mammalians, however multiple laboratories identified these isoforms with a variety of nomenclature (Table 5, Supplemental Figure S6) (Abdul-Ghani et al., 2001; Fo et al., 2006; Ruggiero et al., 2008). Intracellular localization, interacting proteins, and topology were examined for PRAF1-3 in a variety of species, and distinctions were noted. Whereas PRAF1 was localized to Golgi and endosomes, PRAF2 and PRAF3 were found predominantly in the ER (Abdul-Ghani et al., 2001; Fo et al., 2006; Ruggiero et al., 2008). The endosomal localization and interaction with Rab9, is not surprising. Early endosomes are derived from the trans-Golgi network, eventually maturing into late endosomes. Late endosome trafficking back to the trans-Golgi network is dependent upon the activity of Rab9 (Pfeffer, 2011). Additionally as seen with Yipf family members (see above), steady-state localization of a protein does not always correlate with the pattern seen with dynamic trafficking. Hence, the endosomal localization of PRAF1 may not be unexpected.

Further evidence for PRAF family members as regulators of intracellular trafficking was found when other PRAF1-interacting proteins were identified. In addition to interactions with Rab1/3A and VAMP2 (Martincic et al., 1997), PRAF1 was also shown to interact strongly with Rab4B/5A/5C and Rab5/7/9 (Bucci et al., 1999; Sivars et al., 2003), as well as with ζ1-COP and γ-COP proteins (Lee et al., 2011; Abu Irqeba and Ogilvie, 2019). The latter two proteins are members of the COPI coat that facilitates retrograde trafficking from the Golgi to the ER. Other non-Rab interactions were also demonstrated for the PRAF family, including homo- and hetero-dimerization between PRAF2 and PRAF3 (Schweneker et al., 2005; Ruggiero et al., 2008), as well as interactions of PRAF3 with two ER-shaping proteins that both possess hairpin structures, Arl6IP1 and Rtn2B (Liu et al., 2008; Yamamoto et al., 2014). Arl6IP1 was subsequently shown to bind atlastin, though its specific function within the ER is not known. Lastly, it was demonstrated that PRAF1 and PRAF2 were enriched in synaptic vesicles isolated from rat brain (Fenster et al., 2000; Koomoa et al., 2008). More specifically, PRAF1 was demonstrated to interact directly with Piccolo, a multi-domain zinc finger protein that is a novel component of the presynaptic cytoskeletal matrix localized to the active zone of neurotransmitter release. Their interaction occurred via zinc fingers found in Piccolo (Fenster et al., 2000). Similar interactions have not been described for PRAF2 or PRAF3.

A role for PRAF family members in apoptosis has also been elucidated, when it was demonstrated that BCL2A1 (a BCL2 family member) interacted with PRAF1 and inhibited BCL2A1 anti-apoptotic activity (Kim et al., 2019). Such an interaction may be due to conformational changes seen with BCL2 family members during the mitochondrial translocation process. Specifically, apoptotic agonists such as BAX can cause BCL2 family members to adopt a hairpin topology with APH domains, to insert into mitochondrial membranes (Dlugosz et al., 2006; Youle and Strasser, 2008). In this manner, BCL2 family members may transform into MSAPs. Given the propensity of such hairpin-containing proteins to oligomerize and interact, it is not unreasonable to suggest that PRAF1 could interact or regulate the conformational change seen with BCL2 family members, similar to REEP interactions with atlastin described above. It has been demonstrated that PRAF1-3 have a role in regulating apoptosis in several cell lines (e.g. neuroblastoma, myeloid leukemia, and fibroblast cells), however, interactions with PRAF1-3 and BCL2 family members was not elucidated (Kim et al., 2019).

#### 5.2.2 PRAF structure/topology

**FIGURE 5:**
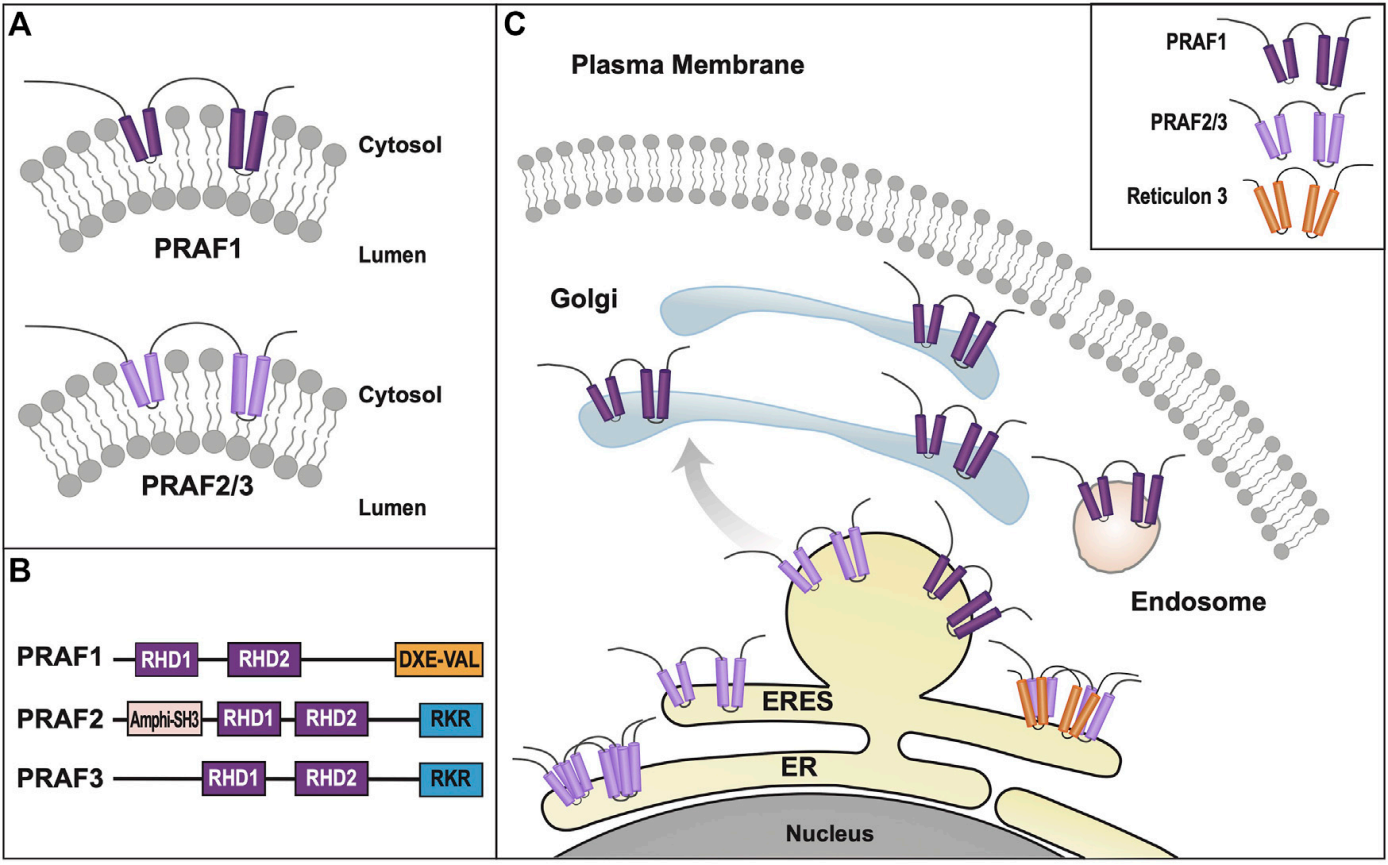
PRAF/Yip3p Family of Proteins. **(A).** Transmembrane topology model of Yip3p and PRAFs based on biochemical analyses (Abdul-Ghani et al., 2001; Lin (J). et al., 2001; Fo et al., 2006). **(B).** PRAF1 and PRAF2/3 have similar topologies but differ in their carboxy termini. Both have conserved YipD domains that most likely form hairpins that insert into the membrane, however they differ by the presence of either a cluster of acidic amino acid residues and a terminal Val (PRAF1) or basic amino acid residues (PRAF2/3). Potential APH domains have not been identified within this family, as has been described in other Yip families. However, an “amphiphysin Src homology 3 (SH3) group’ domain (A-SH3) has been identified in the amino terminus of PRAF2 but not PRAF3 (Lin C.-l. G. et al., 2001). **(C).** Intracellular localization of various PRAF family members and known binding partners is shown within the ER, Golgi, and ERGIC compartments, including intracellular transport vesicles, COPI and COPII. Modeled proteins are not shown to scale relative to their amino acid sequence. ER = Endoplasmic Reticulum, ERES = ER Exit Site, ERGIC = ER/Golgi Intermediate Compartment.

Also, functional domains and specific amino acid regions involved with PRAF function were identified. Computational modeling predicted four transmembrane or hydrophobic regions, with cytoplasmic facing longer amino and shorter carboxy termini (Figure 5A), but no APH domains have been delineated. As well, potential phosphorylation sites within the amino terminus and a potential “amphiphysin Src homology 3 (SH3) group’ domain (Amphi-SH3) within then amino terminus of PRAF2 have been identified (Figure 5B) (Lin J. et al., 2001; Fo et al., 2006). In general, different PRAF isoforms possess amino and carboxy termini of varying lengths and with different specific domains identified, though their role in PRAF function has not delineated. Another interesting distinction between PRAF1 and PRAF2/3 is the presence of a cluster of basic amino acid residues in the extreme carboxy termini of PRAF2/3, whereas PRAF1 instead has a cluster of acidic amino acid residues in the corresponding region (Figure 5B) (Abdul-Ghani et al., 2001).

Further analysis of PRAF structure-function relationships, revealed that the amino terminal portion of PRAF1 was not necessary for Golgi localization, however deletion of either five or ten carboxy terminal amino acids, resulted in an ER localization (Figure 5C) (Liang and Li, 2000; Liang et al., 2004). More specifically, deletion of only the terminal valine residue (Val^185^) or mutation of multiple glutamic acids near the terminal valine resulted in ER retention as well (Jung et al., 2011). Of note, terminal Val residues preceded by such acidic amino acid residues often are binding sites for members of the PSD-95 family of proteins, important for regulation of post-synaptic densities in neurons (Lee and Zheng, 2010). As well, DXE motifs preceding a terminal Val residue have been shown to function as ER exit sequences for ER to Golgi transport (Jung et al., 2007; Zaarour et al., 2009). It was further demonstrated that Val^185^ was necessary for PRAF1 to form homodimers and exit the ER (Liang et al., 2004). By making PRAF1 and PRAF3 chimeras, it was shown that the extreme carboxy terminus of PRAF3 was important for ER localization, and that multiple basic amino acid residues in this region (compared to acidic amino acid residues in PRAF1) were necessary for proper retrieval from the Golgi compartment of multiple cargo proteins (Abdul-Ghani et al., 2001).

#### 5.2.3 PRAF cargo trafficking

Similar to the original identification of REEPs based on their function as GPCR receptor-expression enhancing proteins, PRAF3 was independently identified as a protein that could reduce membrane expression of a glutamate transporter, excitatory amino-acid carrier 1 (EAAC1). It was demonstrated that the interaction between EAAC1 and PRAF3 occurred at the carboxy terminus of EAAC1 (Lin C.-l. G. et al., 2001; Ruggiero et al., 2008). Co-expression of PRAF3 with EAAC1 delayed ER exit of the latter, resulting in an immature pattern of glycosylation, as would be expected for a protein trapped in the ER (Ruggiero et al., 2008). Similar results were seen in Arabidopsis, where expression of certain PRAF isoforms led to ER accumulation of cargo proteins with immature glycosylation (Lee et al., 2011). However, another binding partner for PRAF3, the ER morphogen Rtn2B, could enhance plasma membrane expression of EAAC1 and that the interactions between PRAF3 and EAAC1 for Rtn2B required different domains, so that binding sites did not appear to compete. Thus, it appeared that two separate, but potentially related protein families, could have opposing effects of trafficking of a cargo protein. Specifically, PRAF3 reduced plasma membrane expression of EAAC1, whereas Rtn2B enhanced it, as seen by following glycosylation of EAAC1 as it moved from ER to Golgi to the plasma membrane.

Similarly, other excitatory amino acid transporters (EAAT1-4) were also shown to be regulated by PRAF3 in a manner similar to EAAC1 (Butchbach et al., 2002; Ruggiero et al., 2008). In addition, other GPCRs were identified as potential cargo proteins, whose trafficking from ER to plasma membrane was inhibited by co-expression of PRAF2 or PRAF3 (Table 6). For example, β_2_ and α_2B_ ARs, and D_2_ receptors were shown to be downregulated by PRAF3 expression, and that ER retention correlated with immature glycosylation of the proteins, as seen for EAAC1 (Ruggiero et al., 2008). Other GPCRs were subsequently studied, and it was found that PRAF3 reduced plasma membrane expression of the δ opiate receptor (DOR) and PRAF2 could bind to and regulate membrane expression of the CC chemokine receptor 5 (CCR5) and the GABA_B1_ subunit (Schweneker et al., 2005; Wu et al., 2011; Doly et al., 2016). In all cases, PRAF isoform co-expression led to ER retention and reduced anterograde trafficking to the plasma membrane. In unpublished data, Doly and colleagues also demonstrated that plasma membrane expression of CCR2, CCR7, and 5HT2 receptors also can be downregulated in a similar fashion by PRAF co-expression, however, the PRAF isoforms examined were not described (Doly and Marullo, 2015).

### 5.3 Yip3p/PRAF family summary

Compared to the other Yip families, members of the PRAF family have the strongest connection to Rab cycling (Sivars et al., 2003). It has been shown that members of the family can form homo- or heterooligomers within the ER or Golgi and that they interact with specific Rab and SNARE proteins. More importantly, a specific role for PRAF1 in Rab cycling was demonstrated, namely that PRAF1 can function as a GDI-displacement factor (GDF), to allow for Rabs to return and insert into their cognate membrane (Figure 1). Such a specific role in Rab cycling has not been demonstrated for any other Yip family members, including PRAF2 or PRAF3, and their yeast ortholog Yip3p.

Though specific Rab interactions have been identified for PRAF1, no interacting Rabs have yet been identified for PRAF2 or PRAF3. Aside from Rabs and homo-/hetero-dimerization, only a few other interacting proteins have been identified for the PRAF family. For PRAF1, these include a single SNARE (VAMP2) and a zinc finger containing protein found within presynaptic cytoskeletal matrix of nerve terminals (piccolo). Interestingly, PRAF2 was shown to interact with another ER resident (Rtn2B), as well as a novel hairpin containing protein (Arl6IP1) that also appears to be a potential ER morphogen like REEPs and Rtns. Much like the other Yip families, the PRAF family can interact with Rab and SNARE proteins, and a few novel proteins as well. Unlike REEPs and Yif1A/B, interactions with molecular motors (e.g., dynein, Kif5B) and microtubules have not been demonstrated for PRAF family members.

The different intracellular localizations of PRAF1 (Golgi/endosomes) and PRAF2/PRAF3 (ER) may relate to the Rab proteins with which they interact, though the Rab interacting domain has not been delineated (aside from its localization to the amino terminus). It is intriguing to suggest that the differential localization may relate to the unique carboxy termini of the three PRAF family members (Figure 5B). It has been shown that the terminal Val^185^ and multiple acidic amino acid residues within the extreme carboxy terminus of PRAF1 are important for oligomerization and Golgi localization, however, the ER-localized PRAF2 and PRAF3 isoforms do not possess the terminal valine and they possess multiple basic, not acidic, amino acid residues in their carboxy termini. This significant difference in charge between different family members is similar to that seen with REEP1-4 vs REEP5-6, with respect to net charge seen between the hairpins (discussed above) (Schlaitz et al., 2013).

It remains to be determined if Rab specificity is due in part to specific sequences in PRAF1-3 that interact with Rabs, or possible due to sequences in PRAF1-3 that dictate intracellular localization, or both. If PRAF family members function as GDFs for all Rabs, then the specificity of action must depend on something other than a 1:1 pairing of a specific Rab or Rab subfamily with a specific PRA isoform. Given that there are over 60 mammalian Rab proteins (Stenmark, 2009), PRAF isoform specificity could arise for a Rab subfamily rather than specific Rabs. Interestingly, there is one yeast ortholog (Yip3p), three mammalian isoforms (PRAF1-3) but nineteen PRAF isoforms in *Arabidopsis* divided into 8 clades, which include multiple PRAF2 and PRAF3 orthologs (Alvim Kamei et al., 2008). Similar expanded PRAF families have been seen in other plants (e.g., rice, poplar), so the expansion of the family throughout evolution must have an underlying biological reason.

The exact topology of any PRAF isoform has not been determined biochemically, only by modeling and comparison to other Yip family members. It appears that there are only four hydrophobic/transmembrane domains, unlike the five regions initially identified in Yipf and Yif families, or the two hairpin domains shown for REEPs, Yop1p, and Rtns. The exact membrane topology (i.e., transmembrane vs. hairpin) needs to be determined (Figure 5A). However, the extended amino termini and variable carboxy termini of PRAF1-3 appear to be cytoplasmic as seen for other Yip family members, as predicted by AlphaFold (Jumper et al., 2021; Varadi et al., 2022). However, no APH region has been identified, as found in other Yip family members, but an Amphi-SH3 domain was identified in the amino terminus of PRAF2, which could be relevant for its localization to synaptic vesicles or post-synaptic densities. Lastly, potential phosphorylation sites were identified in PRAF2, but not PRAF1 or PRAF3, however no biochemical characterization of these sites was performed, as done for REEPs.

Similar to REEPs, PRAF2 and PRAF3 (but not PRAF1) have been shown to regulate plasma membrane expression of a variety of GPCRs and neurotransmitter transporters, however they appear to have the opposing effect. For example, co-expression of PRAF3 reduced, whereas REEPs enhanced, membrane expression of several model cargo proteins. Interestingly, Rtn2B, a hairpin-containing ER morphogen, similar in structure to REEP, enhanced EAAC1 expression by enhancing ER exit, whereas PRAF3 expression reduced ER exit of EAAC1. Unfortunately, REEP isoforms were not studied for EAAC1. The interplay of various Yip family members on intracellular trafficking of a single model cargo protein has not been assessed, so one can only speculate as to how these various families may interact to regulate membrane transport of cargo proteins.

## 6 Comparison of yip subfamilies

### 6.1 Cellular and biochemical function

Members of the mammalian Yip family were discovered from a variety of starting points. Some family members were identified based upon the overall homology to yeast Yip proteins and their roles in intracellular membrane trafficking (e.g., Yipf family). Others were discovered based upon their cellular function as regulators of cargo protein trafficking and final cellular localization (e.g., REEP family), and in some cases both starting points intersected (e.g., Yif and PRAF families). However, further research has filled in the gaps from intracellular transport to cargo protein processing and trafficking for some mammalian Yip subfamilies. Despite their evolution from yeast to higher eukaryotes, Yip subfamilies appear to have similar, but possibly subfamily-specific functions in intracellular transport. Overall, there is solid evidence that all four subfamilies and their yeast counterparts share the following findings:

1) Specific interactions occur within and between all four subfamilies and with proteins that comprise the intracellular transport machinery (e.g., Rab/Ypts, COPII vesicles, Sec proteins)
2) They are localized to specific intracellular organelle membranes implying possible specialized functions for different subfamilies
3) Unique protein domains are found within specific subfamilies, that have been shown to be important for their roles as regulators of intracellular transport (e.g., MTB, APH, RHD, YipD, 14-3-3 binding sites)

Through evolution, the mammalian Yip family arose from the corresponding Yeast Yip orthologs. Despite their low amino acid homology, these proteins meet the new definition of a superfamily based upon their common protein domains and topology (though the analysis is incomplete for all subfamilies) (Das et al., 2015). The original cloning of yeast Yip1p and other related yeast proteins arose from research focused on studying Ypt regulation. Biochemical evidence for specific Yip protein interactions has been limited, as reviewed above, however, genetic database analysis (e.g. Rab GTPase trafficking networks (“The Membrome”) has suggested many other potential protein interactions within intracellular trafficking that remained to be explored (Gurkan et al., 2005; Gurkan et al., 2007). So, despite there being missing “pieces” to the Rab/Ypt machinery “interactome” in some Yip subfamilies, it seems apparent that the function of these proteins and their protein partners have been conserved in evolution.

It appears that Yip subfamilies have evolved from the yeast orthologs to encompass more functionalities. For example, REEP1-4 and Yif1B have been shown to bind to tubulin via specific tubulin-binding domains, in fact two such domains have been identified in REEPs, one in the cytoplasmic carboxy terminus and the other between the two RHD domains. In the case of higher vertebrates, binding of these proteins to tubulin aligns Yif1B and REEP1 with the microtubule cytoskeleton in neurons, possibly facilitating the delivery of ER vesicles and cargo to specific neuronal regions. Such domains have not been found in members of the Yipf and PRAF families, however, given the known interaction and oligomerization that can form between different subfamilies (e.g., REEPs, Yif), it would not be necessary that each subfamily have such a domain, as long as a Yip family protein of the complex contained such a domain.

Furthermore, REEP1-4 have evolved to contain multiple potential phosphorylation sites that serve as binding sites for members of the 14-3-3 protein family. Such a region is not found in the original member of the subfamily, Yop1p, nor its closest orthologues REEP5-6. By interacting with 14-3-3 proteins, REEP1-4 have the added functionality of binding to an adapter protein in a phosphorylation-dependent manner, further increasing their complexity of interaction within intracellular trafficking pathways. There are seven known 14-3-3 proteins and they interact with their respective binding sites in a dimeric fashion, so there are many possible combinations of kinases, phosphorylation sites in REEP1-4, and 14-3-3 dimers to explore. Such adapter-protein binding has not been described in other Yip subfamilies, but again, if one member of a heteromeric complex of various Yip proteins can bind to an adapter protein, then the whole complex will be connected to the adapter protein as well. Additionally, complete analysis of potential phosphorylation sites for Yipf, Yif and PRAF families have not been examined, nor the effect of organelle-specific kinase activation on Yip member function.

Analysis of several biochemical and cellular functions of Yip family proteins has shown great overlap across the subfamilies (Table 7). For example, it is clear that all Yip subfamilies (yeast and mammalian) have been shown to interact with proteins of the Rab transport machinery (e.g., Rabs/Ypts, COPII vesicle proteins), so it appears that they play a role in this area of cell biology. However, a functional role for a Yip family member in the regulation of Rabs has only been shown for PRAF1, and possibly Yipf2, when it was shown that they could function as a GDF in Rab cycling. It remains to be seen if PRAF2-3 have a similar biochemical function or if any other Yip family members are GDF or interact with any other aspect of Rab cycling (Figure 1). With respect to cargo transport, specific cargo and regulated cellular trafficking pathways have been identified for members of the REEP, Yif, and PRAF families, but few cargo proteins or cellular pathways have been identified for the largest family, Yipf. Additionally, other interacting proteins such as tubulin and molecular motor proteins have only been shown conclusively for REEP and Yif family members, it remains to be investigated for the Yipf and PRAF families.

**TABLE 7:** Summary of known Yip family member biochemical and cellular properties.

	Membrane topology	SNARE/Rab interacting proteins	Tubulin/Motor interacting proteins	Adapter function	Cargo effects	Rab effects
Yipf	**+/**−	**++**	−	−	**+**	**+**
REEP	**++**	**+**	**++**	**++**	**++**	−
Yif	**+/-**	**+**	**++**	−	**++**	−
PRAF	**+**	**++**	**-**	**++**	**++**	**++**

− = not described to date in the literature.

+/− = conflicting data in the literature.

+,++ = Relative strength of data in the literature.

### 6.2 Membrane localization and the role of APH domains

An important determinant of Yip subfamily function appears to be their membrane localization (Supplemental Figure S7). In general, each subfamily appears to be localized to specific organelles, throughout yeast to mammals and plants. For example, the Yipf and Yif families all localized to the ER - > ERGIC - > Golgi (though some specificity within these compartments have been identified within the family) and REEPs were found in the ER. This simple pattern of localization was not seen for the PRAF family, where it has been shown that PRAF1 localizes to Golgi and endosomes, whereas PRAF2/3 localize to the ER. Some differences may relate to methodology used for localization (i.e., overexpression of epitope- or G/YFP-tagged proteins vs specific Yip antisera and endogenous expression). Despite these differences in technique, the similarities between localization of these proteins within yeast and higher eukaryotes suggests that the data is consistent and therefore specific organelle localization may be relevant to Yip function.

How proteins localize to specific organelles or membranes has often focused on the presence or absence of organelle-specific (e.g. ER) retention or trafficking motifs in cytoplasmic regions of many proteins (Zaarour et al., 2009; Jung et al., 2011). Given the high homology of the hydrophobic/transmembrane regions (e.g., RHD, YipD) within Yip subfamilies and the localization of Yip subfamilies to specific organelles, it suggests that something beyond amino acid trafficking motifs or RHD/YipD domains may be in play. Recently, a comprehensive comparison of transmembrane domains (TMD) from fungi to vertebrates has suggested that TMDs may have organelle-specific properties such as length and composition that may interact with the physical properties of membrane bilayers (Sharpe et al., 2010). It was shown that TMD length increases from Golgi to plasma membrane, in an apparent step-change in bilayer thickness and TMD specificity for an organelle is linked to amino acid residue volume and correlates to changes in lipid asymmetry. Thus, organelle-specific localization of different Yip subfamilies would not be unexpected, as they share a high homology in the hydrophobic/transmembrane domain, within, but not between, subfamilies. Though it appears that Yip family members may possess hairpins and not traditional transmembrane domains, the above hypothesis may still be relevant.

**FIGURE 6:**
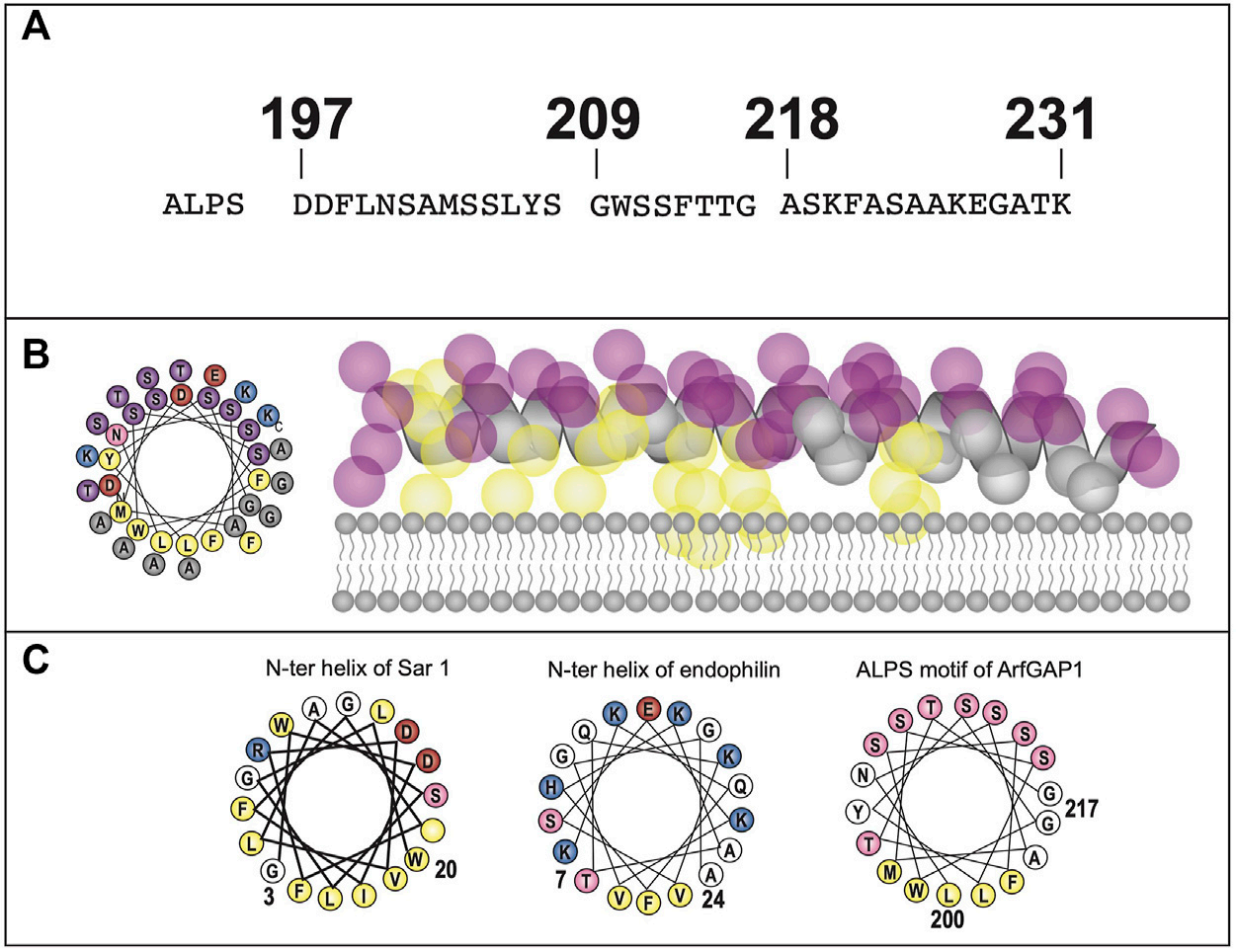
Overview of Membrane Shaping/Sensing APHs. **(A).** Prototypical APH (Amphipathic-Lipid-Packing-Sensor/ALPS) domain from ArfGAP1 is shown in single amino acid code and as a helical wheel diagram (yellow: bulky hydrophobic residues, purple: serines and threonines, gray: glycines and alanines, blue: positively charge residues, red: negatively charged residues) (Modified from Reference (Drin et al., 2007)). The helical wheel was generated using HELIQUEST software (Gautier et al., 2008). **(B).** An alternative atomistic view, demonstrating the backbone arrangement of hydrophobic (yellow) and hydrophilic (magenta) amino acid residues is also shown). Notice the facial separation of hydrophobic and hydrophilic residues which allows for the APH to either sense or associate with membrane lipids (Modified from Reference (Van Hilten et al., 2020)). **(C).** Similar helical wheel diagrams for APHs from other proteins that exhibit organelle membrane-specific localization are shown: Sar1 (ER membrane), endophilin 1 (plasma membrane) and ArfGAP1 (Golgi membrane) (yellow: bulky hydrophobic residues, pink: serines and threonines, white: glycines and alanines, blue: positively charge residues, red: negatively charged residues). It has been postulated that APHs are adapted to different membranes and can either induce (Sar1, endophilin) or sense (ArfGAP1) membrane curvature, depending upon the size of the hydrophobic face as well as the charge distribution within the hydrophilic face (Antonny, 2006). However, no specific amino acid sequence homology has been found to delineate these differences, suggesting that a combination of amino acid composition, spacing, and packing as well as membrane configuration may all play a part in such membrane specialization.

Membrane-shaping domains of various proteins are not limited to transmembrane/hairpin regions that insert into the membrane. Amphipathic α-helix (APH) domains have been discovered in many protein families, including many Yip subfamilies, where they possess “membrane-shaping” or “membrane-sensing” properties (Drin et al., 2007; Bhatia et al., 2009). APH motifs adopt a parallel orientation with the membrane plane due to segregation of hydrophobic and polar amino acid residues on opposite faces of the α-helix (Figure 6A). It appears that the abundance and specific type of polar amino acid residues may render the motif a sensor of membrane curvature, whereas APH domains rich in basic amino acid residues may promote membrane curvature instead. More interesting, it appears that the amino acid composition of APH domains in various proteins may be adapted to organelle-specific membranes of different physicochemical properties, as suggested for Sar1/ER, endophilin/plasma, and ArfGAP1/Golgi membranes respectively (Figure 6B) (Antonny, 2006). Though beyond the scope of this review, it has been suggested that variations within the distribution of hydrophobic and polar amino acid residues within APHs may impart them with different interfacial properties. Such parameters include the size of hydrophobic residues and their density per helical turn; the nature, the charge, and the distribution of polar residues; and APH length (Gimenez-Andres et al., 2018).

Not all alpha-helical hydrophobic domains are transmembrane, such as the eighth alpha helical domain of many seven transmembrane G protein-coupled receptors (GPCRs). It has been shown that this domain is important for proper folding and expression of multiple GPCRs and may function as an APH (Thielen et al., 2005; Sato et al., 2016). Other amphipathic helices have been found elsewhere within GPCRs, where they appear to be important determinants of cell-specific receptor expression. For example, the amino terminus of α2_C_ARs contain a conserved APH, not found in α2_A_ARs, that appears necessary for proper trafficking and targeting of α2_C_ARs to the plasma membrane of neuronal or neuron-like cell lines (i.e. PC12 cells) (Angelotti et al., 2010). Interestingly, transferring only the α2_C_AR APH domain into the α2_A_AR amino terminus, lead to neuronal-specific trafficking of α2_A_ARs, similar to that observed with WT α2_C_ARs (Angelotti et al., 2010). Similar APH domains were identified within the amino termini of α1_D_AR, P_2_X_6_ ligand-gated ion channel, and KChIP4 auxiliary subunit of Kv4 potassium channels, where they also appeared to regulate membrane expression (Hague et al., 2004; Ormond et al., 2006; Tang et al., 2013).

It was originally hypothesized that these GPCR APH domains functioned as either ER-retention signals or possibly interacted with chaperone or other proteins to regulate receptor trafficking, leading to multiple attempts to identify such interacting proteins. An alternative hypothesis would be that APH domains do not interact with other proteins to regulate trafficking, but instead they identify specific membranes via their recognition of membrane lipid composition, to effect final trafficking of cargo proteins within specific membranes or cell types.

Originally, Yop1p was described as having five transmembrane regions, now postulated to be two hairpins, instead of four transmembrane domains. However, the fifth “transmembrane” domain was characterized as an APH domain that aligned parallel to the membrane, and in fact may be important for membrane fusion. Similarly, it was demonstrated that such APH domains are found in other members of the Rtn family, and that similarly placed APH domains were found in all REEP family members (but the data was not shown) (Brady et al., 2015). Such APH domains have also been found in known REEP-interacting proteins, specifically atlastins, where they assist in ER membrane fusion (Moss et al., 2011; Liu et al., 2012; Faust et al., 2015). Recently, it was demonstrated that a carboxy terminal APH domain in a plant RTN (RTN13) was necessary to induce membrane constriction of ER tubules *in vivo* (Breeze et al., 2016). Specifically, deletion or disruption of the hydrophobic/polar face of the APH domain abolished this effect, while retaining its ability to interact and form homo-oligomers via the RHD. Thus, it was proposed that membrane-shaping proteins may rely on APH domains for their function.

Beyond the REEP family, the presence of such APH domains have not been well described for other members of the greater yeast and higher eukaryotic Yip family, though such possibilities exist. Specific APH domains within Yip subfamilies may be responsible for their particular organelle-specific localization. It is possible that organelle-specific membrane composition (e.g., charge density, membrane thickness, lipid composition) may be important determinants of protein localization to a specific organelle. If so, then the RHD/YipD and APH domains may be responsible for differentiating the localization of various Yip subfamilies between organelles. The possibility that these APH domains, along with hairpin motifs, may interact with specific organelle or plasma membrane lipid domains to affect protein cargo trafficking has not been examined. It would be interesting to see if Yip organelle-specific localization could be altered by exchanging APH domains between different subfamilies.

### 6.3 APH domains and hereditary spastic paraplegia

As discussed above, many proteins implicated in the development of HSP (e.g., M1-spastin, atlastin-1, and REEP1) have similar membrane topologies of partial membrane spanning hairpins and APH domains. Similar to other Yip family members, these APH domains may sense or recognize different organelle-specific membranes of different physicochemical properties, as suggested for other proteins (e.g., Sar1, endophilin, and ArfGAP1) (Figure 6B). Recent research on HSP genetics have identified other genes and their encoded proteins as causes of other forms of HSP, specifically proteins involved in lipid/sterol metabolism (Supplemental Table S2). These proteins include DDHD1, DDHD2, PNPLA2, CYP7B1, and CYP2U1 (Blackstone, 2018b).

DDHD1 and DDDHD2 both possess phospholipase/triglyceride lipase activity. Interestingly, DDHD2 lipase activity produced lysophospholipids on the *cis*-Golgi side, which appear to be important for the function and maintenance of the *trans*-Golgi network, as well as regulating Golgi to ER retrograde transport (Morikawa et al., 2009; Sato et al., 2010; Tani et al., 2012; Gonzalez et al., 2013). Disruption of DDHD2 *in vivo* lead to massive neuronal lipid accumulation (Inloes et al., 2014; Inloes et al., 2018). Additionally, DDHD2 may be important for transport of proteins through the Golgi network as well as inducing membrane tubules (Bechler et al., 2012). In a similar manner, other lipid/sterol metabolizing enzymes implicated in HSP (e.g., PNPLA2, CYP7B2, and CYP2U1) may alter membrane composition in the ER and other membrane compartments (Wortmann et al., 2015).

A unifying hypothesis for the multitude of genetic causes of HSP may be that implicated mutant proteins may either alter the structure or form of proteins that contain APH domains (e.g., M1-spastin, atlastin-1, REEP1/2) or alter the lipid composition or structure of ER and/or Golgi membranes (e.g., DDHD1/2, PNPLA2, CYP7B1, and CYP2U1). Such an alteration in membrane lipid structure or composition could prevent proper membrane recognition or insertion of M1-spastin, atlastin-1, and/or REEP1/2 APH domains or hairpins. In either manner, proper structure or transport of proteins could be disrupted, leading to the intracellular neuronal changes observed in HSP.

### 6.4 Yip family comparison with other hairpin containing proteins

Given the homology of the various Yip subfamilies, especially concentrated in the transmembrane/hydrophobic domains, it would be unusual if some subfamilies had a hairpin structure in these regions, while others did not, but prediction of hairpin structure is not straightforward. Based upon experimental and computer modeling data (AlphaFold), a possible unifying structure is beginning to emerge for all Yip family members and other related proteins, hairpin domains with a possible amino or carboxy terminal APH domain buried in or on the membrane (Kranjc et al., 2017). If hairpin structures could be demonstrated for Yipf and Yif members (possibly two sets of dual hairpins as opposed to four transmembrane domains) with a possible fifth alpha helical APH domain, it would be consistent with previous findings and thus would bring a unifying structure/topology to the family. Many other non-Yip proteins involved with intracellular membrane trafficking have also been suggested to have hairpin and APH (or other protein/lipid-interacting domains), instead of simple transmembrane domains. Such proteins include caveolin 1–3 (Cav1-3), reticulon 1–4 (Rtn1-4), flotillin-1/2 (reggie-1/2), protrudin, and FAM134B (Table 8) (Morrow and Parton, 2005; Bauer and Pelkmans, 2006; Voeltz et al., 2006; Hashimoto et al., 2014; Bhaskara et al., 2019).

**TABLE 8:** Other potential membrane-shaping adaptor proteins (MSAPs).

HGNC	Yeast homolog	TM/HP domains	Localization	Interacting proteins
Caveolin 1 (Glenney, 1992)	—	1	PM/Caveola (Ariotti et al., 2015)	—
Caveolin 2 (Scherer et al., 1996)	—	1	PM/Caveola (Ariotti et al., 2015)	—
Caveolin 3 (Biederer et al., 1998)	—	1	PM/Caveola (Ariotti et al., 2015)	—
Rtn1 (Roebroek et al., 1993)	Rtn1p (Geng et al., 2005)	2	ER (Voeltz et al., 2006)	—
Rtn2 (Roebroek et al., 1993)	—	2	ER (Voeltz et al., 2006)	—
Rtn3 (Moreira et al., 1999)	—	2	ER (Voeltz et al., 2006)	—
Rtn4 (Roebroek et al., 1993)	—	2	ER (Voeltz et al., 2006)	—
Protrudin (Shirane and Nakayama, 2006)	—	3	ER (Saita et al., 2009)	Kif5a-c (Chang et al., 2013; Hashimoto et al., 2014)
				Atlastin-1 (Hashimoto et al., 2014)
				REEP1/5 (Hashimoto et al., 2014)
				VAP-A (Saita et al., 2009)
				Rab11 (Saita et al., 2009)
FAM134B (Khaminets et al., 2015)	Atg40p (Mochida et al., 2015)	4	ER (Khaminets et al., 2015)	LC3 (Khaminets et al., 2015)
				GABARAP (Khaminets et al., 2015)

Caveolins are well described proteins, localized to caveolae of the plasma membrane where they serve as scaffolds or adapter proteins, to bring together different signaling molecules (Okamoto et al., 1998). These cholesterol-binding proteins form oligomeric structures that associate and/or induce formation of lipid raft domains called caveolae. Other similarities between caveolins and Yip family members is that they can form homo- and heteromeric complexes and the region of the protein necessary for such formation is the hairpin domains described previously (Das et al., 1999). This finding is similar to oligomerization described above for many of the Yip subfamilies. A region termed the “caveolin-1 scaffolding domain” has been identified in the amino terminal region. This region adopts a β-strand structure and appears to be important for interactions with multiple signaling molecules and cholesterol (Fernandez et al., 2002).

**FIGURE 7:**
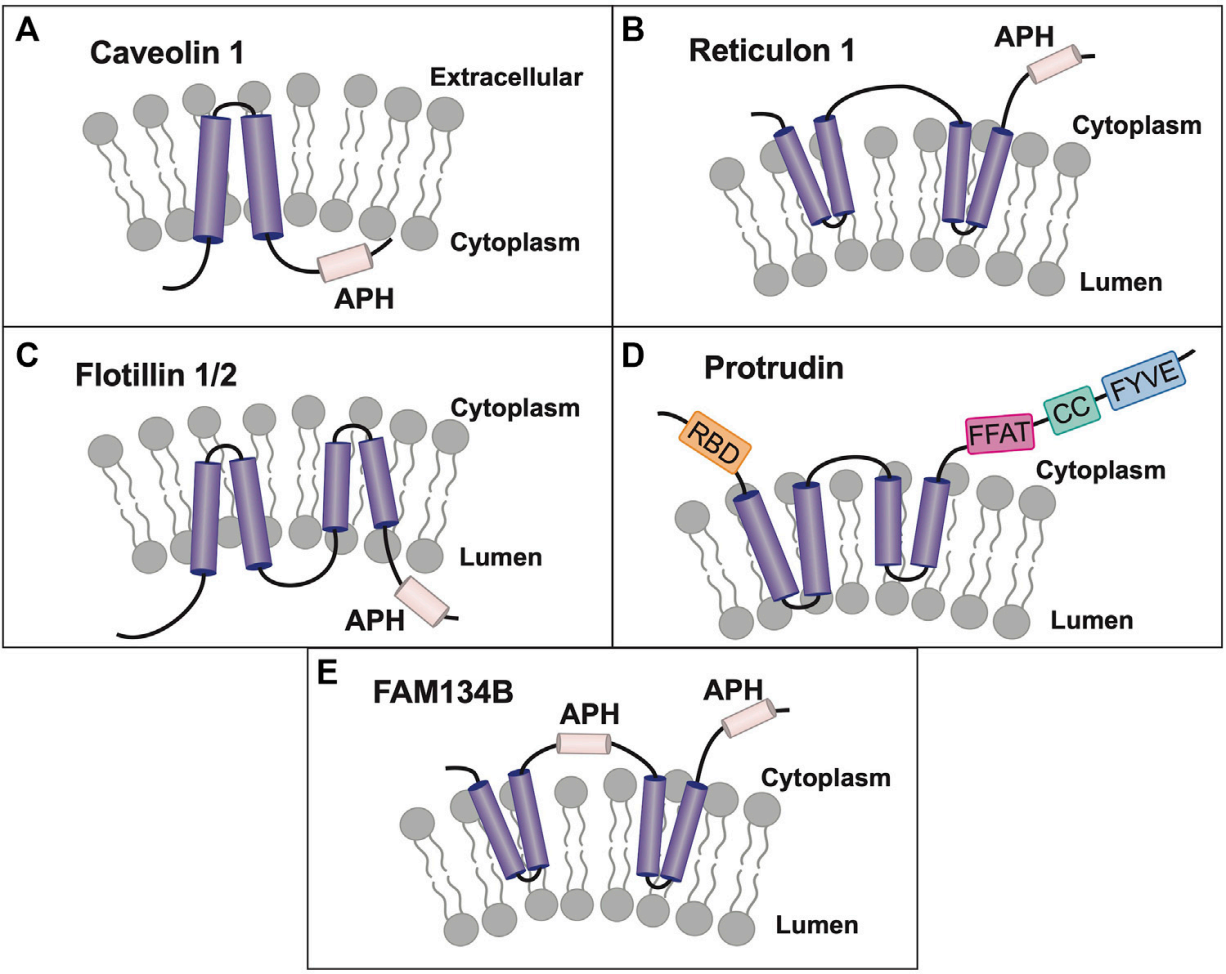
Caveolin and Other Membrane Shaping Adapter Proteins (MSAPs). **(A).** Transmembrane topology of Caveolin-1 (Cav-1). Unlike Yip family members, caveolins possess only a single hairpin domain with a carboxy terminal APH, however they meet all of the criteria to be termed MSAPs (Bauer and Pelkmans, 2006). **(B).** Reticulons share the most homology with REEP/Yop1p, possessing two membrane-inserted hairpin structures and a possible carboxy terminal APH (Shibata et al., 2008; Breeze et al., 2016). **(C).** Flotillin-1/2 are membrane-associated proteins found on the plasma membrane cytoplasmic face. They possess two hairpin structures, found within an amino terminal prohibitin-like domain (PHB) and a carboxy terminal APH (Morrow et al., 2002). **(D).** Protrudin, a binding partner of REEPs and atlastins, also has two hairpin domains that appear to insert into the membrane. However, instead of an APH domain, they possess a FYVE domain that discriminates between different types of phosphoinositides found in lipid rafts (Chang et al., 2013). In addition, it possesses an amino terminal ‘Rab-binding domain’ (RBD) and carboxy terminal ‘coiled-coil’ (CC) and ‘two phenylalanine’s (FF) in an acidic tract’ (FFAT) domains. **(E).** FAM134B, a selective ER-phagy receptor, has been recently demonstrated to possess two pairs of dual hairpin structures, with an APH domain localized between them and a carboxy terminal APH, similar to other Yipf and REEP family members (Bhaskara et al., 2019). Modeled proteins are not shown to scale relative to their amino acid sequence.

When a hairpin predictive model was applied to Cav1 (Figure 7A), it assumed a single hairpin structure with an alpha-helical carboxy terminus that laid parallel to the membrane via palmitoyl groups (an APH domain), reminiscent of the structure described above for Yop1p, except that the later has two hairpin structures and a single APH domain (Spisni et al., 2005). Three caveolin proteins have been identified, Cav1-3, all with similar structural features. Multiple caveolin-interacting proteins have been identified, including heterotrimeric G-proteins, where caveolins appear to function as a GDI or GAP (Figure 1) (Okamoto et al., 1998). As well, Cav1 appears to function as a GDI for Cdc42 (Rho family GTPase) (Nevins and Thurmond, 2006). Additionally, Ha-Ras, Src family tyrosine kinases and endothelial nitric oxide synthase also appear to have specific interactions with various regions of Cav1. Similar to some Yip family members, caveolins appear to function as GDI for a different family of GTPases, possess a hairpin/APH structure, and function as an adapter protein, similar to that described for MSAPs.

The reticulons (Rtns), a large family of proteins that also possess a two hairpin topology, also may function as ER morphogens by altering membrane shape (Shibata et al., 2008; Zurek et al., 2011). Four Rtn genes with multiple splice variants have been described with various amino termini, however, the carboxy second RHD regions are least variable. Overall, the topology of Rtns is similar to that seen with REEPs, with two hairpins, except for longer amino and shorter carboxy termini (Figure 7B). Similar to REEPs, Rtn1p and RTN4A can induce ER tubule formation and directly interact with REEP5 and Yop1 (Voeltz et al., 2006; Shibata et al., 2008; Zurek et al., 2011). More interestingly, it was shown that the plant Rtn homolog Rtn13 possessed a carboxy terminal APH motif, that was necessary for induction of ER tubule constrictions but not necessary for Rtn oligomer formation (Breeze et al., 2016). The presence of similar APH domains in other Rtns remains to be determined. Multiple Rtn-interacting proteins have been discovered for various isoforms. For example, Rtn1 isoforms interact with multiple SNARE proteins including TBC1D20, syntaxin 1, 7, and 13 and VAMP2, a component of the AP-2 adapter complex, Bcl-2, and spastin (Tagami et al., 2000; Iwahashi and Hamada, 2003; Steiner et al., 2004; Mannan et al., 2006; Haas et al., 2007). Rtn3 has also been shown to interact with spastin as well as BACE1 (He et al., 2004; Mannan et al., 2006). Lastly, Rtn3C and Rtn4A/B interact with REEP5, similar to Yop1p interactions with Rtn1p and Rtn2p (Voeltz et al., 2006; Shibata et al., 2008).

Similar to other Yip family members, Rtns have been shown to regulate trafficking of specific cargo proteins. Rtn2B interacts with EAAC1 (excitatory amino acid carrier 1) to enhance its plasma membrane expression, opposing the effect of another Yif family member, PRAF3, which reduces its expression (Liu et al., 2008). Further analysis demonstrated that the first hairpin domain of Rtn2B was necessary for PRAF3 interactions (similar to interactions between other Yip family members), however, the cytoplasmic amino terminal region was necessary for EAAC1 interactions. Therefore, Rtns share many of the same properties as REEPs, including alteration of ER tubule formation and shape, interactions with multiple proteins involved with vesicular trafficking, interactions with multiple Yif family members, and lastly, the ability to regulate plasma membrane expression of specific cargo proteins.

Flotillin-1/2 (Flot1/2) are membrane-associated proteins that have roles in endocytosis, receptor signaling, and interactions with the cytoskeleton (Solis et al., 2007; Otto and Nichols, 2011). Similar to other proteins discussed here, these proteins have been modeled as possessing two hairpin structures, with a short amino terminus and an APH domain in the carboxy terminus (Figure 7C) (Solis et al., 2007). Like caveolins, Flot1/2 are localized to the cytoplasmic face of the plasma membrane, but it has also been found within endosomes, lysosomes, and phagosomes (Morrow et al., 2002; Morrow and Parton, 2005). Both amino termini contain a prohibitin-like domain (PHB), which has been shown to associate with lipid raft domains, most likely via two hairpin structures (analogous to RHD domains in the Yip family) (Morrow and Parton, 2005). Similar to other Yip family members, Flot2 has been shown to directly interact with trafficking machinery proteins, specifically Rab11a and SNX4 (sorting nexin 4), where together they have been shown to affect cargo protein trafficking/recycling (e.g. transferrin receptor, E-cadherin) to the plasma membrane (Solis et al., 2013). Lastly, Flot1 and Flot2 form oligomers, via interactions with the hairpin domains, however, a carboxy terminal alpha helical domain has been identified, which may function as an APH domain (Morrow and Parton, 2005; Solis et al., 2007).

Protrudin, another hairpin containing membrane protein found in the ER and endosomes, may also function as a membrane-shaping adapter protein. Protrudin, a known binding partner of REEPs and atlastins, also appears to have two hairpin domains, but a carboxy terminal APH domain was not delineated (Figure 7D) (Hashimoto et al., 2014). However, it does possess a carboxy terminal FYVE domain, which targets proteins to specific membranes via interactions with membrane-localized phosphoinositides (PtdIns) (Chang et al., 2013). This non-canonical FYVE domain appears to discriminate between various types of phosphoinositides found in lipid rafts, preferring PtdIns(4,5)P_2_, PtdIns(3,4)P_2_. PtdIns(3,4,5)P_3_ over PtdIns(3)P (Gil et al., 2012). Furthermore, it was demonstrated that protrudin functioned as an adapter protein that interacts directly with Rab11a, the motor protein Kif5, and VAP-A, while also enhancing VAP-B, Surf4, and RTN3 interactions with the motor protein Kif5 (Shirane and Nakayama, 2006; Saita et al., 2009; Matsuzaki et al., 2011). These interactions were shown to be important regulators of their transport from the soma to neurites in neuronal cells. Other protrudin-interacting proteins include PDZD8 (a synaptotagmin-like mitochondrial lipid-binding protein), as well as multiple proteins involved with HSP pathogenesis, including myelin proteolipid protein 1, atlastin-1, spastin, REEP1, REEP5, Kif5A-C, and Rtn 1,3, and 4 (Chang et al., 2013; Hashimoto et al., 2014; Elbaz-Alon et al., 2020). Many of these interactions were dependent upon the hairpin domains found within Protrudin. Additionally, it was shown that protrudin contains an amino terminal Rab-binding domain similar in sequence to GDI-α and GDI-β (Shirane and Nakayama, 2006; Chang et al., 2013). Together, protrudin appears to fulfill the criteria necessary to be termed a MSAP.

FAM134B is a selective ER-phagy receptor that regulates the size and shape of the ER, initially modeled with four transmembrane domains, similar to Yipf and Yif family members. However, it was later remodeled utilizing molecular modeling and dynamics simulation as two sets of dual hairpin ‘wedges’ (analogous to RHDs identified in REEPs, RTNs, and Yop1p), but with two APH domains (Bhaskara et al., 2019). The first APH domain was identified between hairpins 2 and 3 and the second APH domain was identified in the carboxy terminus (Figure 7E). The hairpin domains were shown to induce membrane curvature as seen for REEPs and Rtns, and the APH domains were shown to induce asymmetric stretching and compression of the local lipid bilayer, possibly by sensing membrane curvature. FAM134B has been shown to bind to autophagy modifiers LC3 and GABARAP, to facilitate ER degradation by autophagy (“ER-phagy”) (Khaminets et al., 2015). FAM134B oligomerization has been demonstrated and phosphorylation within the hairpin domains by the kinase CAM-K2B promoted oligomerization and ER-phagy (Jiang et al., 2020).

## 7 Membrane-shaping adapter proteins

Yop1p and REEP family members all contain a centrally located dual hairpin structure surrounded by cytoplasmic facing amino and carboxy termini (Park et al., 2010). It is believed that insertion of the dual hairpin into the cytoplasmic face of ER membranes produces high membrane curvature (increasing its surface area), but leaving the cytoplasmic domains available for other possible interactions (Hu et al., 2008). Though not identical in structure, caveolins have a single hairpin that inserts in the cytoplasmic face of the plasma membrane, leading to extreme membrane curvature and creation of caveolae or invaginations of the plasma membrane (Spisni et al., 2005). In addition to caveolins, Yop1p/REEPs, Rtns, protrudin, and FAM134B have been shown to alter membrane shape by insertion of their known hairpin motifs (Spisni et al., 2005; Voeltz et al., 2006; Hu et al., 2008; Zurek et al., 2011; Chang et al., 2013; Bhaskara et al., 2019).

In general, the cytoplasmic termini of caveolins are adapters which can bind multiple protein partners and thus generate signaling complexes for receptor recycling and trafficking (Okamoto et al., 1998). From these studies arose the concept of membrane-shaping adapter proteins (MSAPs), protein families that alter membrane structure and also act as adapters to bind other proteins including plasma membrane proteins (e.g. receptors) and intracellular proteins involved with trafficking (Bauer and Pelkmans, 2006).

MSAPs are defined by their ability to:
1) localize to a specific membrane type(s)
2) alter membrane structure
3) interact with other proteins via specific domains
4) show specificity in their interactions and effects on cargo proteins.

Overall, it appears that many members of the Yip superfamily could meet the definition to be classified as MSAPs, a novel paradigm in membrane organization (Bauer and Pelkmans, 2006). It is clearly evident that REEPs/Yop1p fulfill the criteria to be classified as MSAPs, in that they fulfill all of the criteria listed above. Despite the gaps mentioned above, current research suggests that other Yip family members may represent the largest class of MSAPs, after the caveolin and Rtn families (Bauer and Pelkmans, 2006). This model may represent a new paradigm in membrane organization and may be applicable to the whole Yip family, not just REEPs/Yop1p. Given that APH domains appear to recognize, or “sense” specific membranes based on their lipid composition (which may be important for membrane localization), maybe the term “membrane-shaping adapter protein” (MSAP) should be renamed “membrane-shaping/sensing adapter protein.” Overall, it appears that many protein families may be classified as MSAPs, with the eighteen-member Yip family being the largest, followed by Rtns (four members with multiple splice variants), caveolins (three members), flotillins (two members), and several other individual proteins (e.g., protrudin and FAM134B). Based upon the criteria listed above, other proteins may remain to be discovered or identified as MSAPs.

## 8 Future research directions

If the whole Yip family has been conserved and expanded through evolution, examination of the subfamilies separately and together, can help to fill these gaps and lead to further directions for future research. As extensively detailed above, it is obvious that several unanswered or partially answered questions remain about the role, function, and structure of Yip family members. However, the overlap in current knowledge between different Yip subfamilies, with respect to these questions, implies that common themes most likely are shared. The most compelling lines of research would be to examine the gaps in knowledge between different subfamilies, by utilizing previously applied experimental methods from one Yip subfamily to examine parallel hypotheses in other subfamilies. Additionally, further research would be required to classify Yip family members as MSAPs, specifically do they fulfill all the roles required to be considered MSAPs.

### 8.1 Do all yip family members have similar structure as REEPs/Yop1p?

As discussed above, the first question that needs to be addressed is the membrane topology of the other Yip family members. Current data suggests that most Yip subfamily members share a common two hairpin structure (e.g., REEP, Yif, and PRAF) and two subfamilies possess a fifth alpha helical domain that functions as an APH domain (e.g., REEP, Yif). However, current data suggests that the Yipf family has evolved to consist of five transmembrane domains with the last domain traversing the membrane, making the carboxy terminus intraluminal. It would be of interest to determine why the Yipf subfamily has evolved a different structure and what role this structure may play in its cellular and biological function. Alternatively, if the membrane topology was consistent across all Yip subfamilies (as suggested by AlphaFold), this would aid our understanding of their role and further expand known members of the MSAP family. Bioinformatic analysis of all Yip family members has not been performed, and obvious questions to be answered would be the presence and number of hairpins, as well as the presence and type of APHs (Gimenez-Andres et al., 2018). The methods utilized to examine the structure of REEPs/Yop1p and Yipf family members could be applied to other subfamilies (Voeltz et al., 2006; Kranjc et al., 2017).

### 8.2 Do other yip family members play a role in rab cycling beyond PRAF1?

A common question discussed is whether Yip family members interact with Rabs, by acting as GDFs or some other role (Figure 1). GDFs have been postulated to exist as membrane localizing proteins that displace GDI from cytosolic Rab-GDI complexes, thus returning Rabs to their cognate membranes (e.g., ER, Golgi). Direct proof of such a biochemical interaction was demonstrated, when it was shown that purified PRAF1 could catalytically displace Rab9, but not Rab1 or Rab2, from a Rab-GDI complex onto membranes (Sivars et al., 2003). Therefore, similar experimentation will be required to demonstrate if PRAF2 or PRAF3 serve the same biochemical function and if other Yip family members are GDFs, GDIs, or have other roles in Rab cycling. Indirect cell biological evidence also has suggested that Yipf2 can function as a GDF for Rab5 and Rab22a, however similar biochemical experiments (as described above for PRAF1) remain to be performed (Qi et al., 2019; Wang et al., 2020).

### 8.3 Do other yip family members function as adapters for cargo protein transport?

When cloning REEPs, it was noted that ORs could be immunoprecipitated by REEPs; eventually other GPCR cargos were identified (Saito et al., 2004). In a similar manner, other laboratories cloned cargo interacting proteins based upon yeast two-hybrid screening, identifying other Yip family members. While screening for glutamate transporter (EAAC1) interacting proteins, PRAF2 and PRAF3 were identified. Furthermore, it demonstrated that they interact with other members of the excitatory amino acid transporter family via cytoplasmic interactions with carboxy terminus of PRAF3, suggesting that specific domains within Yip family members interact with specific cargo proteins (Ruggiero et al., 2008). Similarly, Yif1B was shown to interact specifically with a cargo protein, 5HT_1A_R, to target it to specific regions within a neuron (distal dendrites) (Carrel et al., 2008).

Various adapter proteins that interact with Yip family members have been identified, including 14-3-3 family and tubulin within REEPs, however, complete analysis of the Yipf, Yif, and PRAF families has not been undertaken. Given the other domains identified in the latter subfamilies (e.g., amphi-SH3, DXEV) and known binding to molecular motor machinery (e.g., tubulin, Kif5B, dynein), it would not be surprising to find other specific cargo for various Yip family members.

### 8.4 Do all yips function as membrane-shaping adapter proteins?

When examining the literature for only a single subfamily of Yip proteins, their complete role and functional context may be missed due to gaps in knowledge. Examination of the complete Yip family, and its subfamilies, would suggest that they could possess similar membrane topologies and interacting protein domains necessary to move protein cargo through from ER to plasma membrane. Beyond REEPs/Yop1p, it remains to be determined if other Yips meet all of the criteria to be classified as membrane-shaping adapter proteins (MSAPs), specifically if they utilize hairpin domains to insert and thus alter membrane structure and if they function as adapter proteins. By modeling prior research on other Yip subfamilies, it should be possible to determine if all Yipf, Yif, and PRAF family members also fulfill the criteria to be classified as MSAPs.

## 9 Concluding remarks

The emerging concept of Yip family members as MSAPs is an intriguing possibility to explain their myriad roles in cell biology. As MSAPs, Yip family members may have organelle-specific abilities to recognize, insert into (hairpin structures), and otherwise recognize membrane shape and curvature, lipid composition, and possibly membrane charge (APH domain). As well, their adapter function, mediating protein-protein interactions, would serve the purpose as carriers for cargo protein transport from ER, Golgi, and eventually insertion into the plasma membrane. The more compelling question is whether APH domains within other proteins (Yips, GPCRs, and other cargo proteins) dictate localization to specific organelles or plasma membrane subdomains (e.g., dendrite) by their ability to recognize membrane composition or possibly specialized lipid domains or rafts. In this manner, precise membrane localization of a protein may be truly “membrane-delineated” rather than directed by protein-protein interactions.

The Yip family of proteins obviously have a major role in cell biology, specifically as important regulators of intracellular transport of proteins from the ER to the plasma membrane. A significant impediment to further study of Yip family members has been the inconsistent nomenclature, thus making direct comparisons across species tedious and difficult to interpret. Additionally, Yip subfamilies within a single species have not been investigated with similar methods to examine topology, function, and binding partners, leading to incomplete data, and understanding of their biochemical and cellular functions. Partial analysis of individual Yip members or Yip subfamilies, without comparison to the larger Yip family (e.g., Yipf, REEP, Yif, PRAF) made it appear that these proteins had a multitude of somewhat disconnected biochemical and cellular roles. Originally, the larger Yip family appeared to have a cacophony of function within a cell. By using the more commonly accepted nomenclature, this review has attempted to clarify the literature and make prior published research easier to review in context. In this way, the apparent harmony of cellular and biochemical structure, function and interactions of the larger Yip family and its subfamilies hopefully is more evident. It is my intent that this review serve as a resource for new research directions examining the larger Yip family of MSAPs.

## Supplementary material

Supplementary Figure S1 Phylogenetic Analysis of Human Yip Family Members. Protein phylogenetic analysis of all eighteen mammalian Yip family members performed by Creative Proteomics. The horizontal axis represents the distance or dissimilarity between different clusters/proteins, whereas the vertical axis represents the clusters/proteins. Joining of two clusters is represented on this graph by splitting a horizontal line into two horizontal lines with the short vertical bar representing the distance (dissimilarity) between the two clusters.

Supplementary Figure S2 Yipf Family Multiple Sequence Alignment. Amino acid alignment of Yipf1-7. GenBank and HGNC names and total amino acid number are listed on the left. Amino acid types are color coded by type of amino acid: Hydrophilic Basic—Red, Hydrophilic Acidic—Purple, Hydrophilic Uncharged Polar—Green, Hydrophobic Aromatic/Aliphatic—Blue. Conservation and consensus sequences are shown. Alignments and final outputs were created using EMBL-EBI Multiple Sequence Alignment and Jalview (Waterhouse et al., 2009; Madeira et al., 2022).

Supplementary Figure S3 AlphaFold Prediction of Membrane Topology for Yipf/REEP/Yif/PRAF Protein Families. Human Yipf1, REEP2, Yif1A and PRAF1 structures predicted by AlphaFold. For an interactive view, visit https://alphafold.ebi.ac.uk (Jumper et al., 2021; Varadi et al., 2022). Yipf1 https://alphafold.ebi.ac.uk/entry/Q9Y548. REEP2 https://alphafold.ebi.ac.uk/entry/Q9BRK0. Yif1A https://alphafold.ebi.ac.uk/entry/O95070. PRAF1 https://alphafold.ebi.ac.uk/entry/Q9UI14.

Supplementary Figure S4 REEP Family Multiple Sequence Alignment. Amino acid alignment of REEP1-6. GenBank and HGNC names and total amino acid number are listed on the left. Amino acid types are color coded by type of amino acid: Hydrophilic Basic—Red, Hydrophilic Acidic—Purple, Hydrophilic Uncharged Polar—Green, Hydrophobic Aromatic/Aliphatic—Blue. Conservation and consensus sequences are shown. Alignments and final outputs were created using EMBL-EBI Multiple Sequence Alignment and Jalview (Waterhouse et al., 2009; Madeira et al., 2022).

Supplementary Figure S5 Yif Family Multiple Sequence Alignment. Amino acid alignment of Yif1A/B. GenBank and HGNC names and total amino acid number are listed on the left. Amino acid types are color coded by type of amino acid: Hydrophilic Basic—Red, Hydrophilic Acidic—Purple, Hydrophilic Uncharged Polar—Green, Hydrophobic Aromatic/Aliphatic—Blue. Conservation and consensus sequences are shown. Alignments and final outputs were created using EMBL-EBI Multiple Sequence Alignment and Jalview (Waterhouse et al., 2009; Madeira et al., 2022).

Supplementary Figure S6 PRAF Multiple Sequence Alignment. Amino acid alignment of PRAF1-3. GenBank and HGNC names and total amino acid number are listed on the left. Amino acid types are color coded by type of amino acid: Hydrophilic Basic—Red, Hydrophilic Acidic—Purple, Hydrophilic Uncharged Polar—Green, Hydrophobic Aromatic/Aliphatic—Blue. Conservation and consensus sequences are shown. Alignments and final outputs were created using EMBL-EBI Multiple Sequence Alignment and Jalview (Waterhouse et al., 2009; Madeira et al., 2022).

Supplementary Figure S7 Intracellular Localization of Yip Superfamily of MSAPs. Intracellular localization of various Yip superfamily members and known binding partners is shown within the ER, Golgi, and ERGIC compartments, including intracellular transport vesicles, COPI and COPII. Modeled proteins are not shown to scale relative to their amino acid sequence. ER = Endoplasmic Reticulum, ERES = ER Exit Site, ERGIC = ER/Golgi Intermediate Compartment.
