# Supplementary figures and images for: Exploring the eukaryotic Yip and REEP/Yop superfamily of membrane-shaping adapter proteins (MSAPs): A cacophony or harmony of structure and function?
Source: Front Mol Biosci. 2022 Aug 19;9:912848. doi: 10.3389/fmolb.2022.912848 (PMC9437294; doi:10.3389/fmolb.2022.912848)

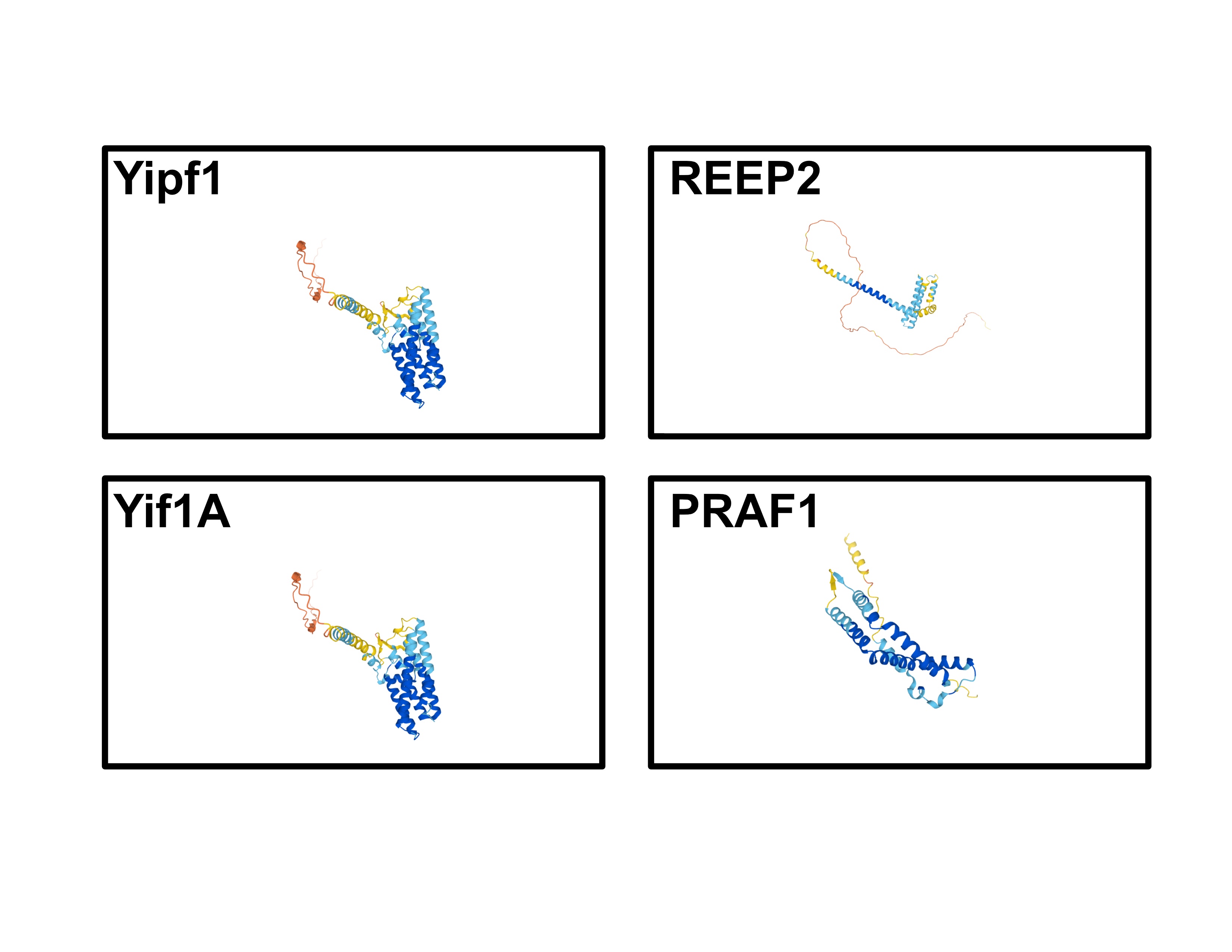

Supplement: Supplementary file 1 [file Image3.JPEG]

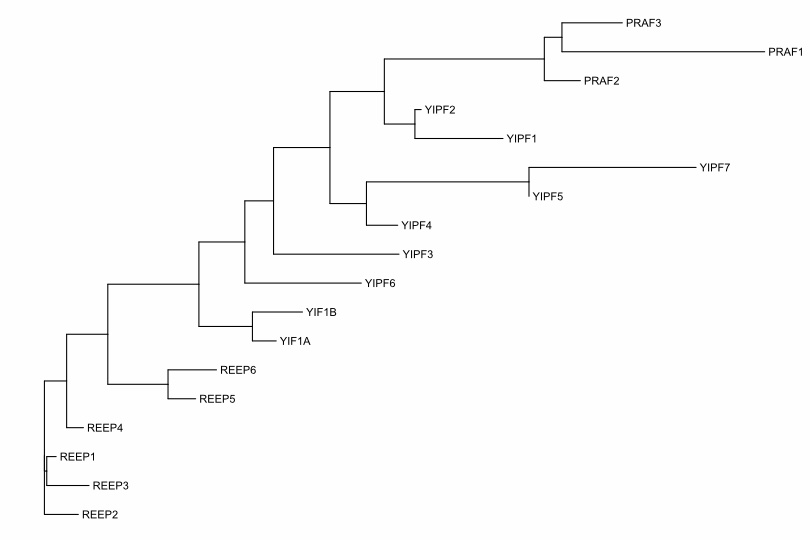

Supplement: Supplementary file 3 [file Image1.JPEG]

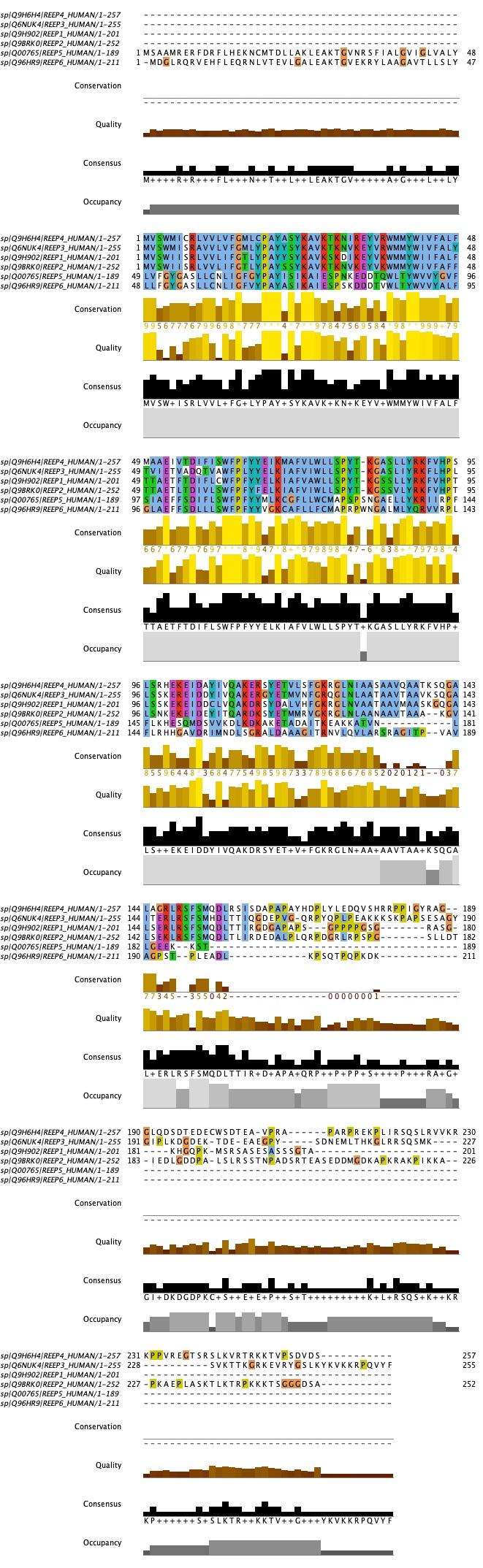

Supplement: Supplementary file 4 [file Image4.JPEG]

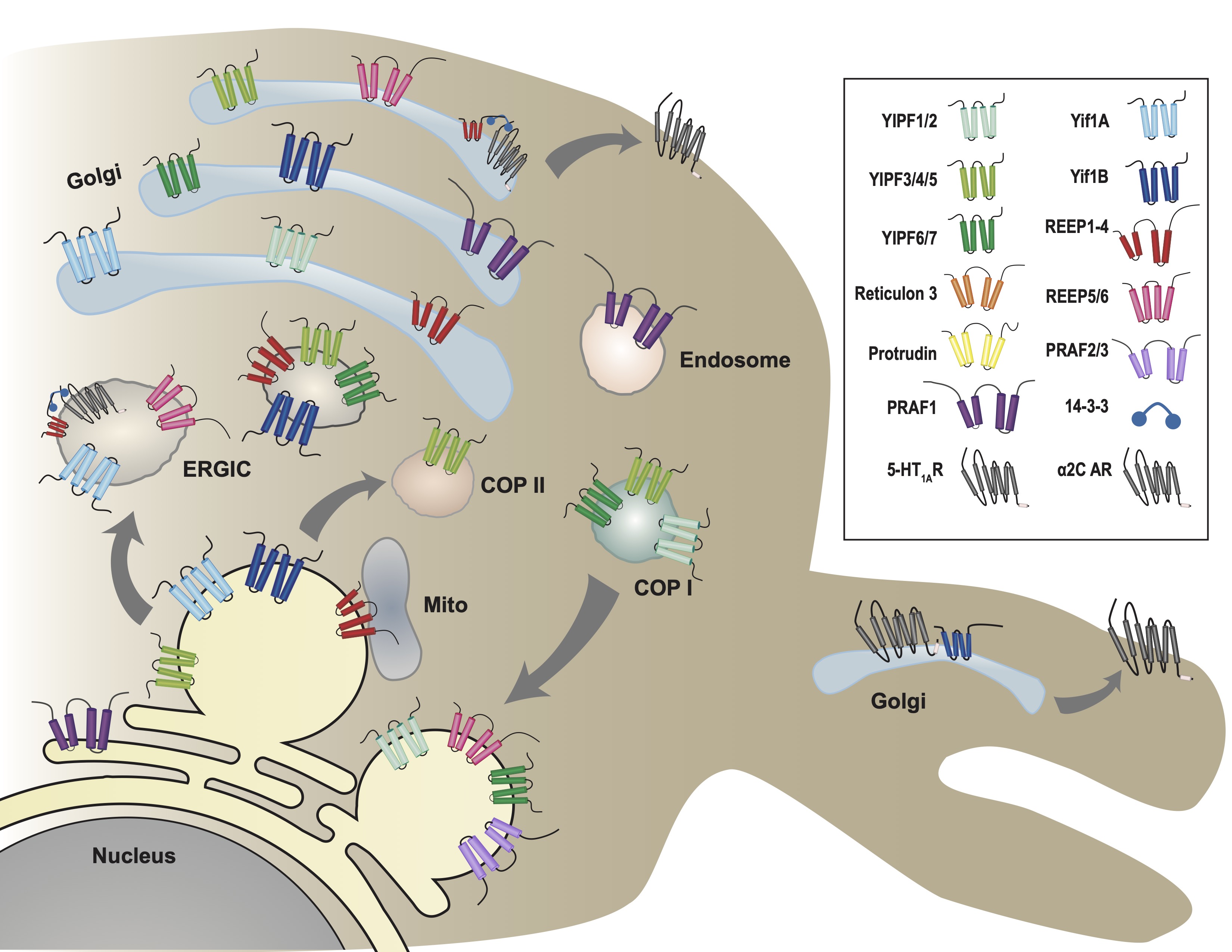

Supplement: Supplementary file 5 [file Image7.JPEG]

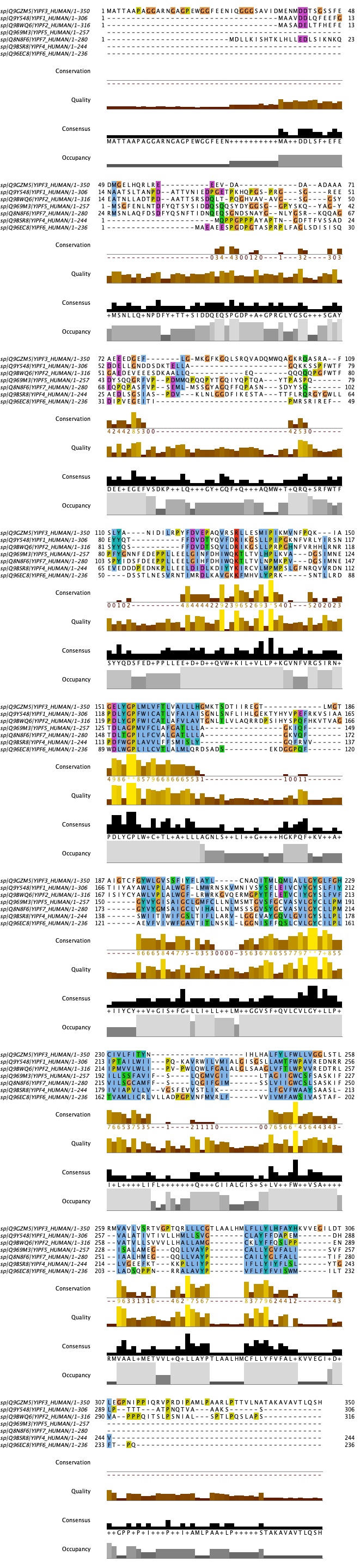

Supplement: Supplementary file 6 [file Image2.JPEG]

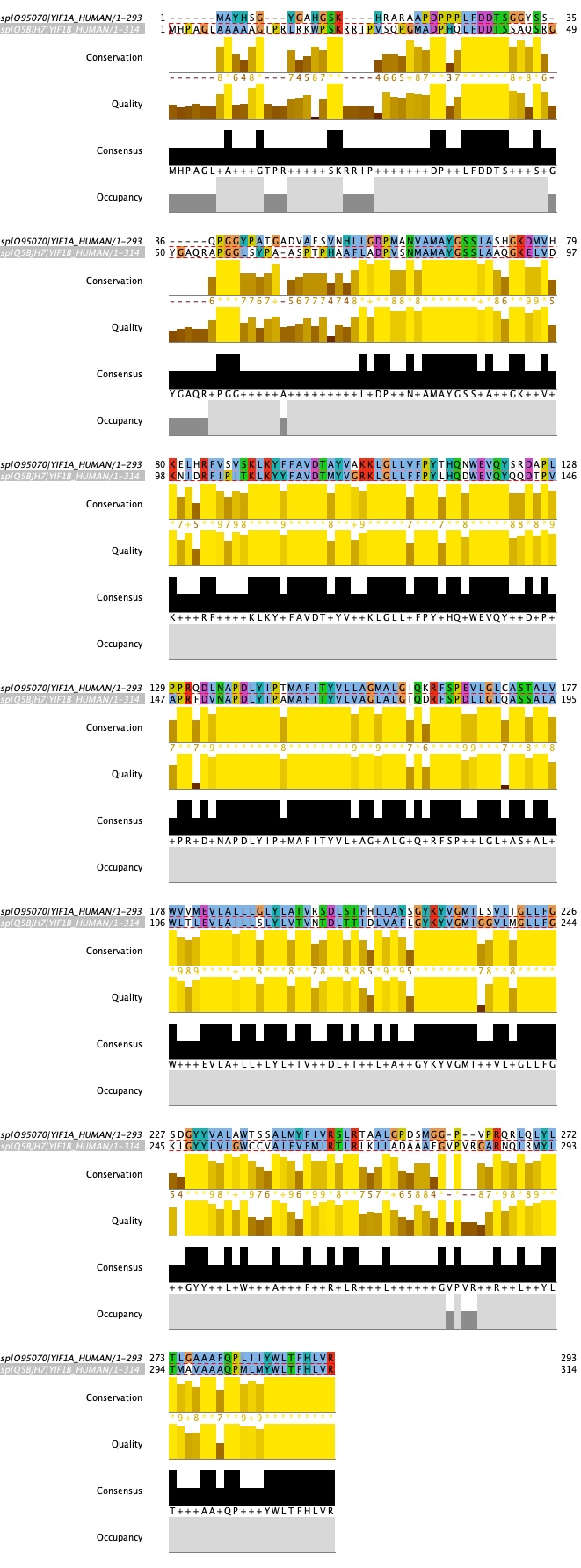

Supplement: Supplementary file 7 [file Image5.JPEG]

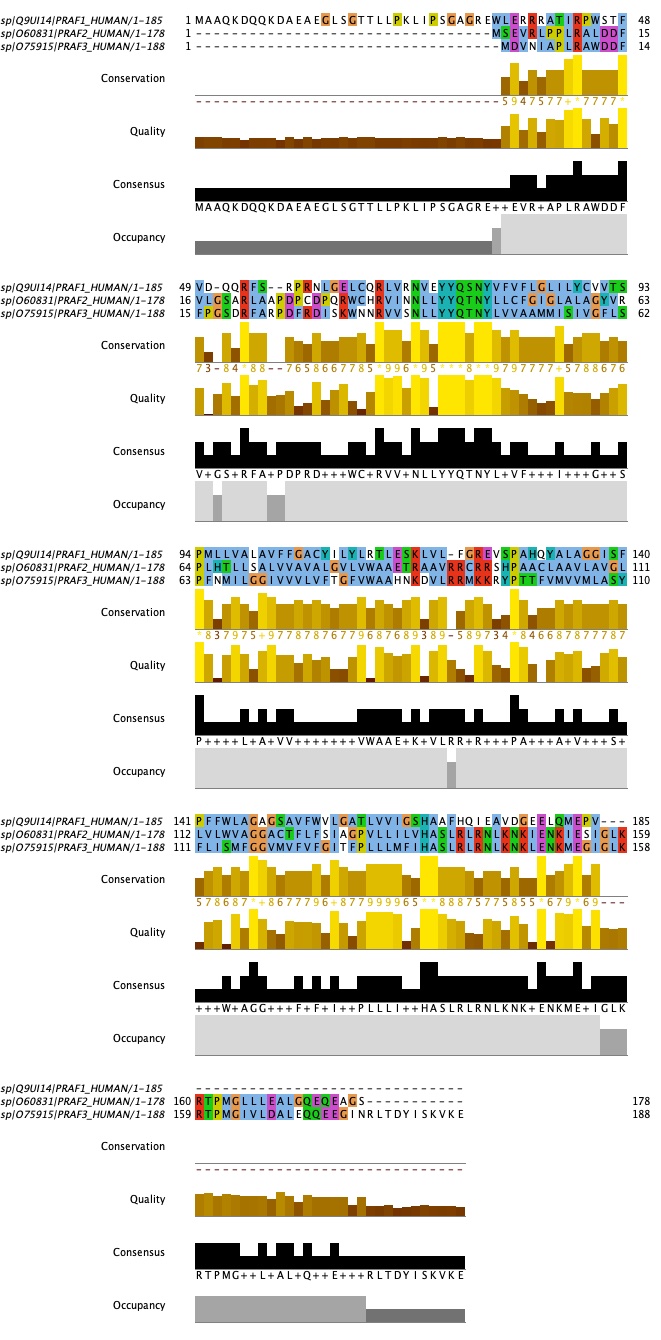

Supplement: Supplementary file 9 [file Image6.JPEG]
