# Supplementary material for: Exploring the eukaryotic Yip and REEP/Yop superfamily of membrane-shaping adapter proteins (MSAPs): A cacophony or harmony of structure and function?
Source: Front Mol Biosci. 2022 Aug 19;9:912848. doi: 10.3389/fmolb.2022.912848 (PMC9437294; doi:10.3389/fmolb.2022.912848)
Supplement: Supplementary file 2 [file Table1.DOCX]

**Supplemental Table II: Hereditary Spastic Paraplegia (HSP) Genes Implicated in Lipid/Sterol Modification**

| **Gene** | **Protein Name** | **Cellular Function** |
| --- | --- | --- |
| **DDHD1** (Tani et al., 2012) | Phospholipase A1 | phosphatidic acid metabolism, membrane traffic |
| **DDHD2** (Bechler et al., 2012) | Phospholipase A1 | phosphatidic acid metabolism, membrane traffic |
| **PNPLA6** (Wortmann et al., 2015) | Neuropathy target esterase | phospholipid homeostasis |
| **CYP7B1** (Tsaousidou et al., 2008) | Cytochrome 7B1 | cholesterol metabolism |
| **CYP2U1** (Apparsundaram et al., 2001; Dhers et al., 2017) | Cytochrome 2U1 | long-chain fatty acid metabolism |
